# Influence of Ultrasound on the Characteristics of CaP Coatings Generated Via the Micro-arc Oxidation Process in Relation to Biomedical Engineering

**DOI:** 10.1021/acsbiomaterials.3c01433

**Published:** 2024-03-19

**Authors:** Balbina Makurat-Kasprolewicz, Marcin Wekwejt, Anna Ronowska, Grzegorz Gajowiec, Marlena Grodzicka, Stefan Dzionk, Agnieszka Ossowska

**Affiliations:** † Department of Materials Science and Technology, Gdansk University of Technology, 80-233 Gdańsk, Poland; ‡ Department of Biomaterials Technology, Gdansk University of Technology, 80-233 Gdańsk, Poland; § Department of Laboratory Medicine, Medical University of Gdańsk, 80-210 Gdańsk, Poland; ∥ Faculty of Chemistry, Nicolaus Copernicus University in Toruń, 87-100 Toruń, Poland; ⊥ Department of Manufacturing and Production Engineering, Gdansk University of Technology, 80-233 Gdańsk, Poland

**Keywords:** plasma electrolytic oxidation, ultrasound, titanium, biomedical application, implants, bone regeneration

## Abstract

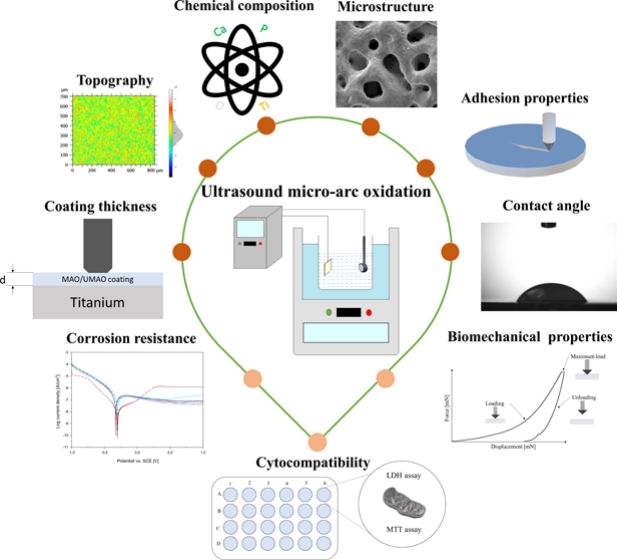

Over the past decade, bone tissue engineering has been at the core of attention because of an increasing number of implant surgeries. The purpose of this study was to obtain coatings on titanium (Ti) implants with improved properties in terms of biomedical applications and to investigate the effect of ultrasound (US) on these properties during the micro-arc oxidation (MAO) process. The influence of various process parameters, such as time and current density, as well as US mode, on the properties of such coatings was evaluated. Novel porous calcium-phosphate-based coatings were obtained on commercially pure Ti. Their microstructure, chemical composition, topography, wettability, nanomechanical properties, thickness, adhesion to the substrate, and corrosion resistance were analyzed. In addition, cytocompatibility evaluation was checked with the human osteoblasts. The properties of the coatings varied significantly, depending on applied process parameters. The US application during the MAO process contributes to the increase of coating thickness, porosity, roughness, and skewness, as well as augmented calcium incorporation. The most advantageous coating was obtained at a current of 136 mA, time 450 s, and unipolar rectangular US, as it exhibits high porosity, adequate wettability, and beneficial skewness, which enabled increased adhesion and proliferation of osteoblasts during in vitro studies. Finally, the conducted research demonstrated the influence of various UMAO process parameters, which allowed for the selection of appropriate Ti implant modification for specific biomedical utilization.

## 1 Introduction

Due to frequent road traffic accidents, falls from a height, a sedentary lifestyle, and an aging population (and consequently increased osteoporosis prevalence), bone tissue engineering has been gaining increasing popularity as a field of science in recent years.^1−3^ Repairing and regenerating broken bones are indispensable to restore the organism’s full functionality.^4^ Among the many biomaterials used for this purpose, Ti can be distinguished. It exhibits many remarkable properties, such as excellent biocompatibility, high specific strength, good fatigue resistance, high chemical stability in the complex environment of human body fluids, good corrosion resistance in the aggressive chemical environment of the human body, and low allergenicity.^5,6^ However, despite being relatively well developed, the implantology of Ti materials still faces difficulties, such as inadequate mechanical properties and lack of osteogenic activity.^7−9^ Those problems contribute to implant surgery failures, which may result from metallosis or stress shielding. Consequently, implant loosening may be due to insufficient bone integration and/or fibrous tissue production.^10−13^

Therefore, scientists around the world attempt to develop innovative implant materials and/or modify their surfaces to (i) reduce the risk of stress shielding occurrence, (ii) increase corrosion resistance and consequently extend the in vivo lifespan of the implant, and (iii) stimulate healing and improve osseointegration of the implant with the surrounding tissue.^7,14^ Surface modifications can be accomplished by using a variety of surface treatments. These include thermal spraying,^15^ ion implantation,^16^ physical vapor deposition,^17^ chemical vapor deposition,^18^ electrophoretic deposition,^19^ anodic oxidation, and micro-arc oxidation (also called plasma electrolytic oxidation).^20^ The MAO is a simple electrochemical process characterized by a relatively short duration. It enables extensive adjustment of parameters (voltage, time, current density, etc.) to obtain ceramic coatings with superior properties. Generally, the MAO coatings are porous, and their micron/submicron hierarchical structure mimics the natural bone architecture.^21^ It can facilitate implant anchorage in human bone tissue.^22^ Furthermore, the MAO coatings exhibit high adhesion to the substrate, improve the corrosion resistance of the biometals, and utilize the alteration of mechanical properties (including hardness and Young’s modulus).^23,24^ Finally, the chemical composition of the coating can be manipulated by simply changing the chemical reagents in the electrolyte.^25^ Concerning biomedical applications, this process is usually performed in solutions containing various Ca- and P- forms to impart increased osteogenic activity of biomaterial.^26−30^ The aforementioned various calcium and phosphorus compounds used in the MAO process deserve special attention because, among the microelements, Ca and P are the two main components of hydroxyapatite, which strengthens the mechanical properties of natural bone.^31^ In recent years, many researchers have focused their attention on the surface modification of metallic implants using MAO and electrolytes with different calcium and phosphorus sources.^32−34^ In many cases, it utilized the generation of coatings with enhanced corrosion and wear resistance as well as bioactivity.^35−41^ It was found that the content of Ca and P in the coatings is influenced by many factors such as (i) concentration of compounds in the electrolyte, (ii) current density during the process, (iii) ion source, (iv) process voltage, and (v) treatment time.^32,40,42^

Despite all the advantages mentioned above, the MAO process is also associated with disadvantages such as (i) accumulation of the mullite phase around the discharge channels, which may deteriorate the hardness of the coating, and (ii) nonuniformity of the microstructure, which may result in the formation of locally reduced corrosion resistance and variable compactness.^43−45^ As a result, these defects can lead to premature failure of the implant at the weak points of the coating in the aggressive environment of body fluids.^45,46^ Therefore, the ultrasound micro-arc oxidation (UMAO) process is derived from the MAO method. The use of UMAO is associated with the same benefits as those of the MAO process. However, US can increase the coatings’ thickness, cohesiveness, and microhardness, modify their microstructure and phase structure, and decline the breakdown potential during oxidation.^22,47,48^ In addition, considering the mechanical effects of the US, it can be stated that their usage can enhance the coating’s uniformity and even distribution of elements.^45^ To date, ultrasound as a novel auxiliary technology in the MAO process has not been scrutinized in detail. Several attempts have been made to create ceramic coatings on metallic substrates such as magnesium and its alloy,^49−53^ aluminum and its alloy^43,47,48,54^ as well Ti and its alloy.^22,45,55^ It was found that the use of ultrasound on aluminum alloy increased the thickness of MAO coatings and favored phase transformation from γ-Al_2_O_3_ to α-Al_2_O_3_.^54^ In the case of Ti-6Al-4 V, it was noted that the US influenced the morphology, phase composition, wear resistance, and corrosion properties of MAO coatings.^56^ This is related not only to the use of ultrasound but also to the electrolyte components and process parameters. Zhang et al.^45^ performed the UMAO process using an electrolyte containing Na_2_Cu-EDTA, calcium acetate, and sodium dihydrogen phosphate. They obtained a uniform, porous, and cytocompatible coating with an increased corrosion resistance of the Ti biomaterial.

Concerning the abovementioned reports, the authors performed the UMAO process on commercially pure Ti using an electrolyte containing calcium acetate hydrate and β-glycerophosphate disodium salt pentahydrate as sources of calcium and phosphorus, respectively. To the best of our knowledge, such chemicals have not yet been used in the UMAO process on Ti. In addition to regular studies evaluating the impact of ultrasound mode (sinusoidal or unipolar rectangular) on the microstructure and chemical composition of coatings, the authors focused on improving material properties, concerning biomedical applications. The modifications were examined for their potential impact on topography, mechanical properties, cytocompatibility, and corrosion resistance. Our work discloses that the UMAO process is a promising method to generate functional surfaces of biomedical materials with improved cytocompatibility and performance. This type of surface treatment can be used to modify commercially pure Ti implants dedicated for, e.g., (i) partial joint resurfacing of the knee or hip (especially as an acetabular, femoral, or tibial component),^57,58^ (ii) craniofacial reconstruction,^59^ and (iii) spinal interbody fusion.^58,59^

## 2 Materials and Methods

### 2.1 Sample Preparation

Commercially pure Ti (ASTM grade 2; Bibus Metals LLC, Dabrowa, Poland) discs with a diameter of 14 mm and a height of 3.7 mm were cut from a homogeneous rod. The substrates were polished using #120, #400, #600, #800, and #1200 grid SiC abrasive papers. Then, the specimens were ultrasonically cleaned and degreased with methanol (p.a.; STANLAB, Lublin, Poland), 2-propanol (p.a.; EUROCHEM BGD, Tarnów, Poland), and distilled water for 10 min each. After ultrasonication, the specimens were dried in ambient air.

### 2.2 Coating Preparation

**Figure 1:**
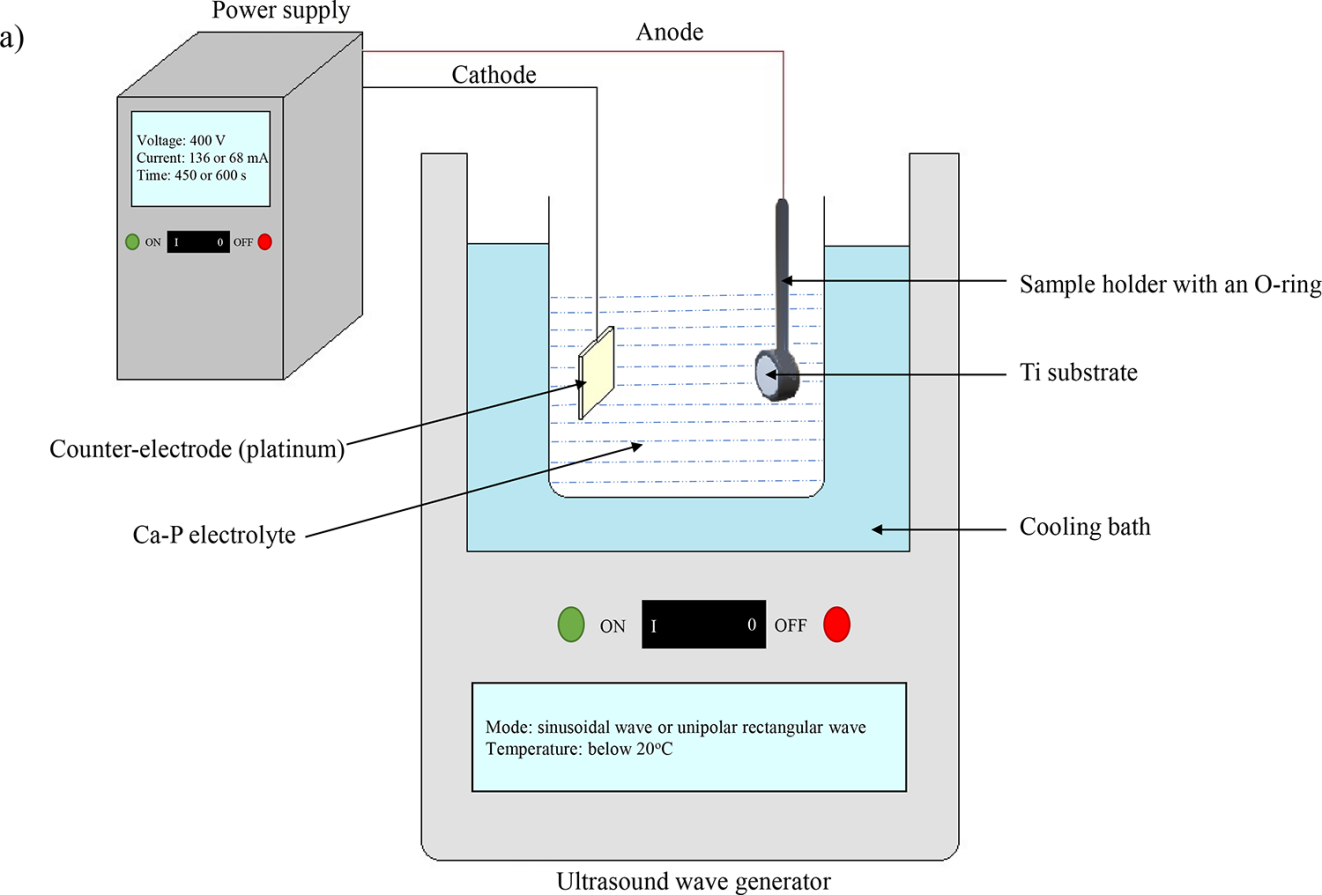
Scheme of the micro-arc oxidation and ultrasound micro-arc oxidation processes. During the MAO process, the ultrasound wave generator was switched off. When the UMAO process was conducted, the ultrasound wave generator was switched on.

The micro-arc oxidation (MAO) and ultrasound micro-arc oxidation (UMAO) processes were performed with a DC power supply (MR 1000020, B&K Precision Corporation, Yorba Linda CA, USA) under a constant voltage of 400 V and a constant current of 68 mA (current density of 60 mA/cm^2^) for 450 and 600 s, and a constant current of 136 mA (current density of 120 mA/cm^2^) for 450 and 600 s. Unlike the conventional MAO process, UMAO was treated with an ultrasound wave generator (FSF-010S, ChemLand, Stargard, Poland) using two operating modes: sinusoidal wave or unipolar rectangular wave (Figure S1) with a frequency of 40 kHz and a power of 80 W. The configuration of the workstation is shown in Figure 1, and detailed designations of MAO and UMAO coatings formed with different parameters are given in Table 1. MAO and UMAO processes were carried out in an aqueous electrolyte containing 14 g/L calcium acetate hydrate Ca(CH_3_COO)_2_ (CAH) (99%; Thermo Fisher Scientific, Fair Lawn, NJ, USA) and 3 g/L β-glycerophosphate disodium salt pentahydrate C_3_H_7_Na_2_O_6_P·5H_2_O (β-GPNa) (≥97%; Merck KGaA, Darmstadt, Germany) as a source of calcium and phosphorus ions, respectively. Ti samples were used as the anode, and the cathode was a platinum electrode (Figure 1). Each Ti disc was fixed onto a holder with an O-ring. Since heat energy is released during the MAO and UMAO processes, the glass container was kept in a water-cooling bath below 20 °C. After treatment, the surfaces were washed with ultrapure water and dried.

**Table 1:** Labels of MAO and UMAO Coatings Formed under Different Conditions

group	label	voltage [V]	current [mA]	time [s]	ultrasounds
—	Ti	—	—	—	—
136_450	136_450_n	400	136	450	
	136_450_sin				sinusoidal
	136_450_rec				unipolar rectangular
136_600	136_600_n			600	
	136_600_sin				sinusoidal
	136_600_rec				unipolar rectangular
68_450	68_450_n		68	450	
	68_450_sin				sinusoidal
	68_450_rec				unipolar rectangular
68_600	68_600_n			600	
	68_600_sin				sinusoidal
	68_600_rec				unipolar rectangular

### 2.3 Experimental Section

#### 2.3.1 Microstructure Analysis

The surfaces of the uncoated Ti sample and samples modified via the MAO and UMAO processes were examined by using a field emission scanning electron microscope (JEOL JSM-7800 F, JEOL Ltd., Tokyo, Japan). The images were analyzed using a secondary electron detector (SED) at a 5 kV acceleration voltage. The average percentage of the open surface porosity and average pore size of the investigated MAO and UMAO coatings were calculated using ImageJ software on SEM micrographs.

#### 2.3.2 Chemical Composition

The elemental compositions of the samples were determined using an X-ray energy-dispersive spectrometer (Edax Inc., Mahwah, NJ, USA; accelerating voltage = 15 kV) attached to the above-mentioned scanning electron microscope.

#### 2.3.3 Topography

Specimens were examined using a noncontact 3D optical profilometer with a confocal technique (S neox 090, Sensofar, Barcelona, Spain). The assays were performed using a Nikon – EPI 20X objective with a magnification of 20.0000. Based on research using SensoMAP Standard 9 software, the statistical parameters for the tested samples according to ISO 25178 were analyzed.

#### 2.3.4 Coating Thickness

The thickness (*d*) of MAO and UMAO coatings (*n* = 10) was measured by the nondestructive coating thickness gauges (FMP 10–20, Helmut Fischer GmbH, Berlin, Germany). The NC/NF mode dedicated to electrically nonconducting coatings on nonferrous metal base material was used.

#### 2.3.5 Adhesion Properties

The adhesion strength of the MAO and UMAO coatings (*n* = 5) was estimated by NanoTest Vantage (Micro Materials, Wrexham, UK) using the Berkovich three-sided pyramidal diamond with an apex angle equal to 124.4°. The test parameters were as follows: scratch load 0–400 mN, loading rate 2.0 mN/s, scan velocity 4 μm/s, and scratch length 800 μm. The loading was linear and continuous. Scratch tests enable assessment of the adhesive and cohesive damage caused to the coating during the research. This study evaluated two parameters: L_c_, the force at which failure occurs, and F_c_, for which the coating undergoes complete delamination. L_c_ was identified by the first differences in the topography before and after the scratch test. The F_c_ was measured by observing scratch test data, i.e., friction force and surface topography, before and after tests. During the scratch test, isolated damage can be ignored;^26,60^ therefore, optical microscopy (UC50, Olympus, Tokyo, Japan) was used to determine where the coating delamination began.

#### 2.3.6 Surface Wettability

The contact angle (CA) measurements with the falling drop method examined the surface wettability (*n* = 5). An optical tensiometer (Attention Theta Life, Biolin Scientific, Espoo, Finland) was used, and demineralized water was a liquid droplet dropped on the samples. The volume of the liquids was about two μL/sample. Each measurement lasted 10 s and was carried out at room temperature. The contact angle analysis was performed by using the OneTennsion program (Biolin Scientific, Espoo, Finland).

#### 2.3.7 Biomechanical Properties

Nanomechanical properties of MAO and UMAO coatings (*n* = 15) were determined using a nanoindenter (Alemnis AG, Thun Switzerland) with Berkovich three-sided pyramidal diamond with an apex angle equal to 124.4°. The tests were performed with a maximum force of 50 mN, loading and unloading times equaled 20 and 15 s, respectively, and the cycle had a 5 s dwell at a maximum load. As a result of the nanoindentation tests, hardness (*H*), maximum indent depth, and Young’s modulus (*E*) values were obtained using the Oliver-Pharr method^61^ based on the results analysis program. To convert the reduced Young’s modulus into Young’s modulus, a Poisson coefficient of 0.28 was assumed for the coatings.^62^

#### 2.3.8 Corrosion Assays

Corrosion tests of samples were performed with a potentiostat/galvanostat (Atlas 0531, Atlas Sollich, Gdansk, Poland) using a three-electrode system (*n* = 3). The results were analyzed directly and independently using AtlasCorr05 software, calculating the corrosion current density and corrosion potential based on Tafel extrapolation and drawing polarization curves. The sample was the working electrode, the platinum rod was the counter electrode, and a saturated calomel electrode (SCE) was employed as the reference electrode. During the test, all electrodes were immersed in 100 mL of Ringer’s solution (Merck KGaA, Darmstadt, Germany), used as the test fluid for many implants.^63^ The solution temperature was kept at 37 °C. First, the open circuit potential (OCP) values were determined. Then, the corrosion curves were recorded by using the potentiodynamic method. The measurements were performed for potential ranging from −1.0 to +1.0 V at a potential change rate of 0.3 mV/s. Using Tafel extrapolation, the values of corrosion current density (*j*_corr_), zero current potential (*E*_*j*=0_), and polarization resistance (*R*_pol_) were determined with the software AtlasLab (Atlas-Sollich, Rębiechowo, Poland). The samples’ corrosion rate (CR) and protection efficiency (PE) were calculated using the obtained current density values (for details, see Protocol S1).

#### 2.3.9 Cytocompatibility

The experiments of in vitro cytocompatibility of obtained samples were conducted with a human osteoblast cell line (hFOB 1.19, RRID: CVCL3708; ATCC, USA). Unless otherwise noted, the reagents were purchased from Merck KGaA (Darmstadt, Germany). hFOB cells were cultured in a 1:1 mixture of Ham’s F12 Medium and Dulbecco’s Modified Eagle’s Medium (no phenol red, DMEM/F-12, Gibco), supplemented with 0.3 mg/mL Geneticin (G418) and 10% Fetal Bovine Serum (10%) at 34 °C in a humidified atmosphere with 5% CO_2_. The cells (8 × 10^4^) were seeded in a drop (100 μL) directly on the samples (*n* = 4; surface area = 1.54 cm^2^), and then, after their adhesion (∼4 h), a medium was added (up to 2 mL) to the holes. The study was conducted for 3 days. The culture medium after experiments was used for the lactate dehydrogenase (LDH, fractional (S)-lactate: NAD+ oxidoreductase) release and the MTT (3-(4,5-dimethylthiazol-2-yl)-2,5-diphenyltetrazolium bromide) assays. Detailed methodology for determining cytocompatibility is provided in Protocol S2.

#### 2.3.10 Cell Adhesion and Proliferation

To calculate cell proliferation on the coatings, the cells (7 × 10^4^) prepared as described above (2.3.9) were seeded on the samples and incubated for 24, 72, and 120 h in standard conditions. The rate of cellular adhesion was performed with the use of MTT assay (see Protocol S2) after chosen periods of time. The final product of MTT reduction formazan was measured spectrophotometrically at a wavelength of 590 nm and a reference value of 690 nm. Results were expressed as relative absorbance (absorbance at 590 nm minus absorbance at 690 nm).

In order to evaluate cell adhesion, the cells (1 × 10^4^) were prepared as described in Section 2.3.9. Herein, they were seeded on the samples and incubated for 24, 72, and 120 h. After each tested incubation period, the medium was removed, and the samples were washed three times with 1 mL of phosphate-buffered saline (PBS). Then, the buffer solution was removed, and the cells were fixed with 0.5 mL of 3.7% paraformaldehyde (PFA) for 15 min at room temperature. Subsequently, PFA was removed, and samples were washed three times with 1 mL of PBS. Afterward, the surfaces of samples were supplemented with 0.5 mL of 0.1% Triton-X 100 in PBS for 5 min. Thereafter, Triton-X 100 was removed, and samples were washed three times with 1 mL of PBS. Then, samples were stained with 0.3 mL of 0.001% FITC phalloidin and incubated for 20 min at room temperature without light. Following that, FITC phalloidin solution was removed, and samples were washed three times with 1 mL of PBS in order to wash out the unbound dye. Eventually, prepared samples were fixed with a Histomount reagent (National Diagnostics, USA) and covered with coverslips. Microscopic observations were performed using a fluorescence microscope (Nikon Eclipse E200, Nikon Instruments Ins., USA) at 505 nm wavelength.

### 2.4 Statistical Analysis

Statistical analysis of the data was performed with commercial software (SigmaPlot 14.0, Systat Software, San Jose, CA, USA). The Shapiro–Wilk test was used to assess the normal distribution of the data. All the results were presented as mean ± standard deviation (SD) and statistically analyzed with a one-way analysis of variance (one-way ANOVA). Multiple comparisons versus the control group between means were performed using the Bonferroni *t* test with statistical significance set at *p* < 0.05.

## 3 Results and Discussion

### 3.1 Microstructure Analysis

**Figure 2:**
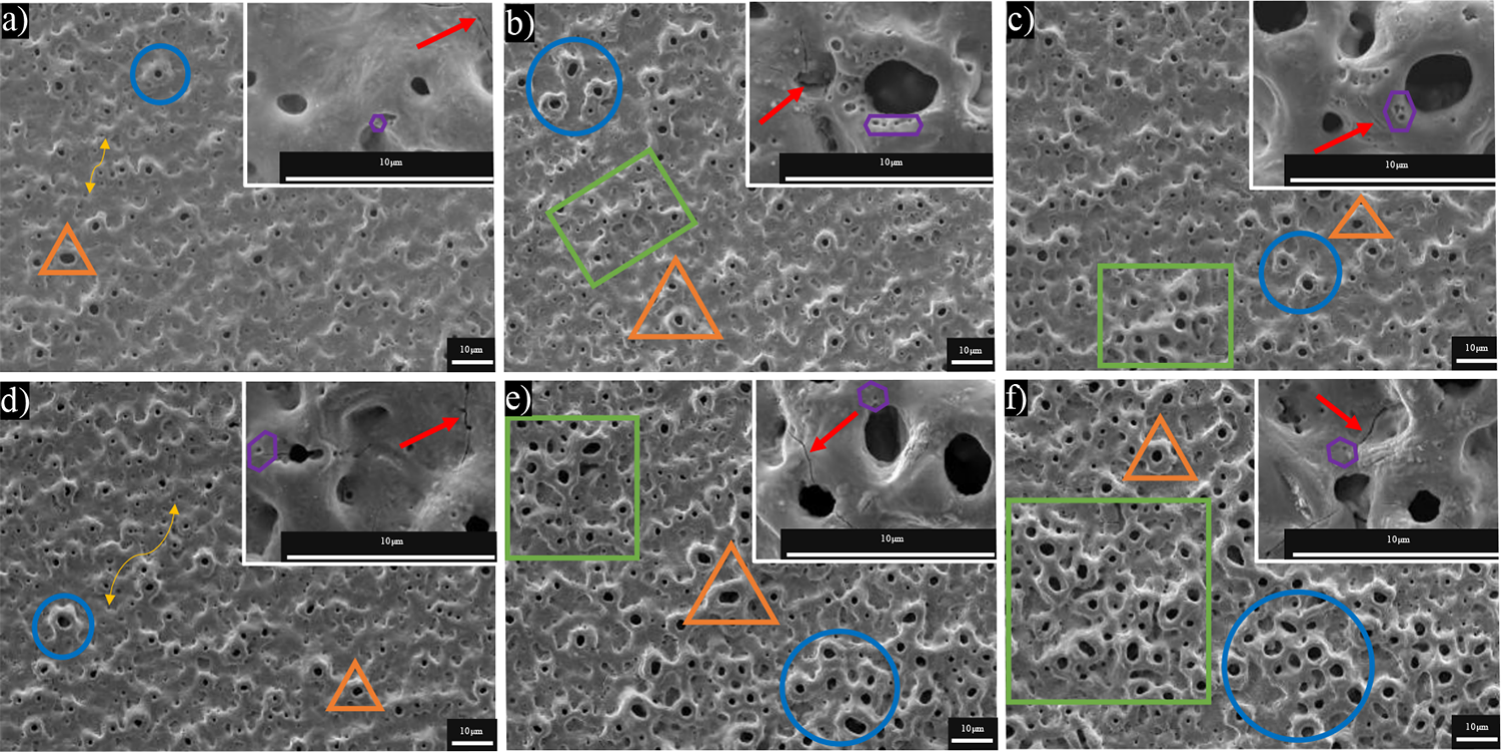
Surface morphology of (a) 136_450_n, (b) 136_450_sin, (c) 136_450_rec, (d) 68_450_n, (e) 68_450_sin, and (f) 68_450_rec coatings. Porous coatings were obtained, and their structure depended on the parameters used. Legend: green quadrangle–net formation; blue oval–volcano-like pores; red arrow–microcrack; yellow double arrow–plane supermicron pores; violet hexagon–submicron pores; orange triangle–micropores. The surface morphologies of the remaining samples are presented in Figure S2.

**Figure 3:**
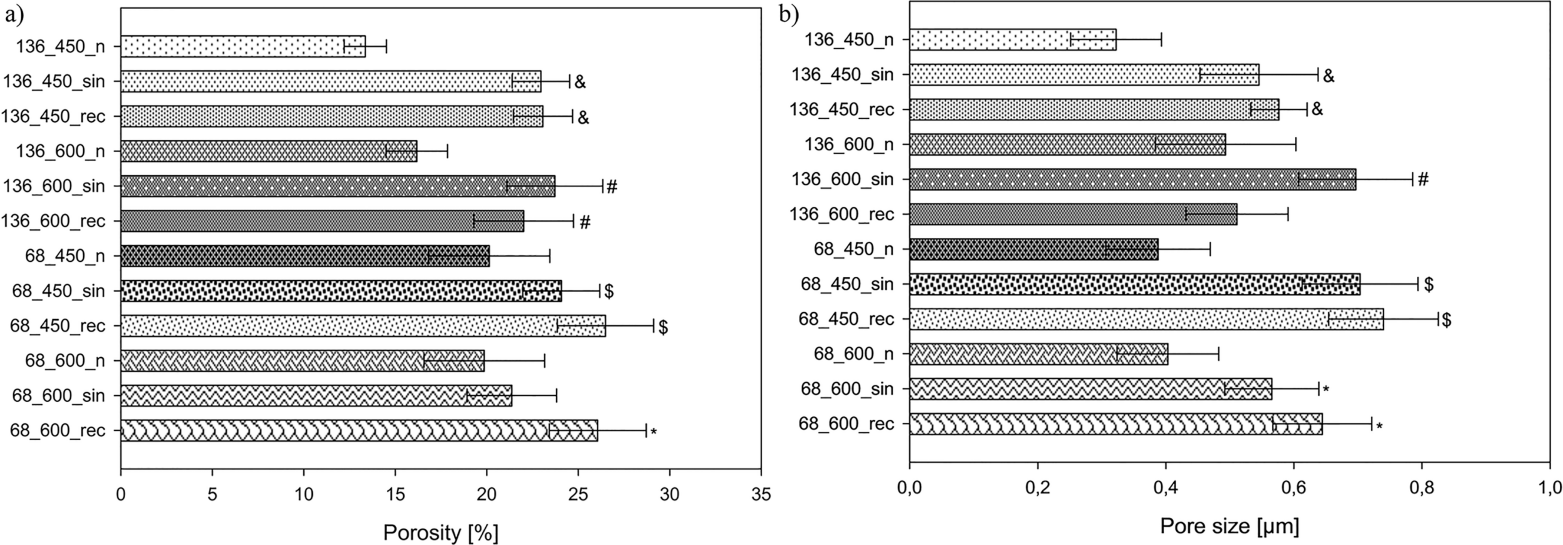
(a) Average porosity and (b) average pore size of the MAO and UMAO coatings formed on Ti in different conditions. UMAO coatings exhibit greater porosity and pore size compared to MAO coatings obtained with the same process parameters. For all analyses, *n* = 9; data are expressed as means ± SD; &, #, $, and *: statistically significant difference between the samples in the groups (136_450, 136_600, 68_450, and 68_600, respectively), as compared to the MAO sample in each group (*p* < 0.05).

SEM studies enabled the analysis of the microstructure of the coatings, and ImageJ software utilized the calculation of the average pore size and porosity of the coatings. Figures 2 and S2 show SEM images of MAO and UMAO coatings formed under different process parameters (Table 1). All coatings, both MAO and UMAO, were porous, rough, and highly folded, which is consistent with the results obtained by many researchers.^7,48,64,65^ Notably, the coatings after the UMAO process are characterized by extensive net formation and a more significant number of volcano-like pores, compared to the coatings obtained with the same parameters due to the MAO process, where mostly plane supermicron pores were structured. Besides, more micropores and submicrometer pores were formed on the UMAO coatings than on the MAO coatings. ImageJ software analysis revealed that applying ultrasound contributes to developing surfaces with higher porosity and larger pores (Figure 3a,b, respectively). The average porosity varied from 21.37 to 26.49% for UMAO coatings and 13.36–20.14% for MAO coatings. Besides, the average diameter of pores for UMAO coatings varied from 0.51 to 0.74 μm, while for MAO coatings, it ranged from 0.32 to 0.49 μm. Porosity and average pore size were higher for UMAO coatings than for MAO coatings obtained with the same process parameters, which was also observed by Qu et al. for magnesium alloy.^49^ The formation of larger macropores may be associated with an increase in the intensity of micro-arc discharges under the influence of ultrasounds, which create acoustic cavitation with a high energy level.^66^ On the other hand, the average pore size for all coatings in our work did not exceed 0.8 μm, which seems to be a relatively small value because in the literature, it is stated that the pore size should be in the range of 100–400 μm to achieve a compromise between the transport needs of cells and nutrients and the mechanical strength of the porous structures. However, this size refers to the macropores and not to the interconnective pore channels.^67^ In addition, it has been shown that submicron and nanosized pores promote increased adhesion of bone cells with the surface of biomaterials, thus creating a more substantial formation of a tissue-implant interface.^68^ For example, Correa et al.^69^ for MAO conducted at 400 V on Ti-15Zr-xMo alloys received coatings with average size 2.8–4.5 μm pores, depending on the Mo content (x). On the other hand, Dziaduszewska et al.^26^ performed the MAO process at 400 V with the same electrolyte concentration as Correa et al.^69^ and received pores of average size 5.7–8.4 μm, depending on the current values. Nonetheless, the process was performed on Ti-13Zr-13Nb Ti alloy, and the substrate influenced the growth of ceramics coatings.^7^ The small average pore size in our study’s MAO and UMAO coatings is associated with many submicron pores that develop due to oxygen penetration.^45^ Furthermore, microcracks appeared on all the coatings, which may be formed due to thermal stress, rapid condensation of the molten compound with the simultaneous working of the cold electrolyte, or rapid transition of oxides from amorphous to crystalline form.^26^ Qu et al.^49^ noted that using US during the MAO process can counteract the formation of cracks. In our study, no significant differences in the occurrence and size of cracks on the coatings were observed, and their formation may have resulted from the application of high voltages and current densities. Moreover, the power of the applied US influences the intensification of the process. The high power of ultrasound leads to the mechanical and cavitation effects, lowering the critical value of the MAO voltage and causing the formation of a large number of electrical sparks in the vicinity of the sample.^49^ Perhaps the use of less power ultrasound would enable the elimination of microcracks.

### 3.2 Chemical Composition

**Figure 4:**
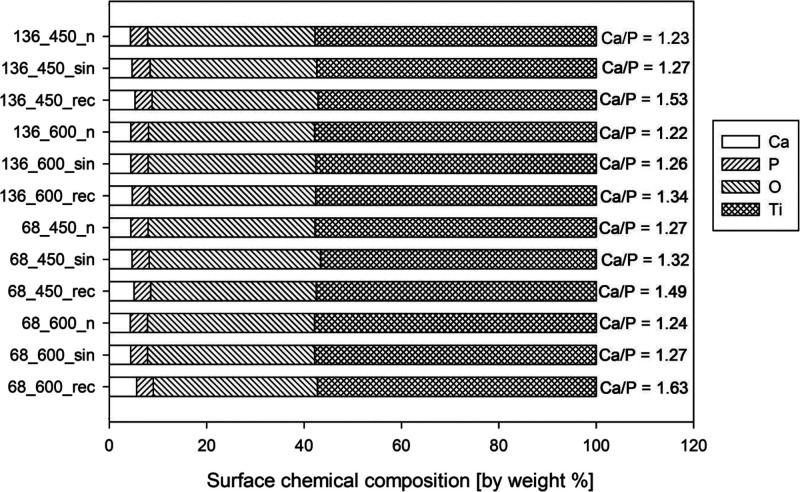
Surface chemical composition (by weight %) and the calcium to phosphorus ratio in the analyzed UMAO and MAO coatings were measured by energy-dispersive X-ray spectrometry EDS. All coatings consisted of calcium, phosphorus, oxygen, and titanium.

EDS microanalysis of MAO and UMAO coatings revealed that the chemical composition of the coatings depends on the process conditions (Figure 4). MAO and UMAO coatings consist of Ca and P from the electrolyte, O from severe anodization, and anodized to severe anodization with Ti from the substrate. The use of ultrasound in each group increased the Ca/P ratio. Simultaneously, calcium and phosphorus contents also increase in UMAO coatings compared to MAO coatings (with the same group), which was previously stated also for magnesium alloy.^49^ However, the phosphorus amount for all samples remained similar and ranged from 3.41 to 3.69 by weight %. The most remarkable changes were noticed in incorporating calcium (ranging from 4.33 to 5.59 by weight %). The use of ultrasound contributed to an increase in the content of this element in the coating, while the use of the sinusoidal mode had a more minor influence on the incorporation than the use of the unipolar rectangular mode. According to the characteristics of these modes, unipolar ultrasound has a maximum amplitude for a more protracted process time than sinusoidal ultrasound, which may contribute to such changes in composition since the more significant the amplitude, the greater the energy carried by the wave.^70^ Generally, ultrasound penetration through the electrolyte causes rapid pressure changes, which consequently force more intense movement of the fluid, and thus, calcium and phosphate ions are incorporated into the coatings.^71,72^ The higher the ultrasound power, the more intense is this process. The Ca/P ratio in UMAO coatings increases compared to MAO coatings, indicating that ultrasound more easily activates the reaction.^47^

### 3.3 Topography

To quantitatively describe the surface topography of samples, the average values (from three measurements) of the roughness (Sa), the kurtosis (Sku), the skewness (Ssk), the maximum peak height (Sp), the maximum valley depth (Sv), isotropy (Is), the core height (Sk), the reduced peak height (Spk), and the reduced valley depth (Svk) were scrutinized. These values were measured using the following parameters: L-filer (λc) — Gaussian, 0.25 mm; S-filter (λs) **—** Gaussian, 2.5 μm. The results are shown in Tables 2 and S1, and the surface topography maps of the samples are presented in Figure S3. The arithmetic mean deviation of the samples after surface modification was much higher than that of the uncoated sample. The average roughness (Sa) of the MAO coatings ranged from ∼0.49 to ∼0.52 μm, while the UMAO coatings ranged from ∼0.57 to ∼1.14 μm. Using ultrasound during the MAO process increased the roughness of the coatings in each of the studied groups, which is consistent with the results obtained by other researchers.^73^ In addition, using bipolar rectangular ultrasound resulted in rougher surfaces than in the case of sinusoidal ultrasound with the same process parameters. It can be related to the fact that with unipolar rectangular ultrasound, the ultrasonic wave shows the maximum amplitude for a longer time than a sinusoidal wave.^70^ Therefore, the unipolar rectangular ultrasound process is more intense. However, the Sa parameter does not enable differentiation of the distinctive features of the surface topography as surfaces with different functional features may have the same Sa parameter value, although they present different surface characteristics. Therefore, the Sa parameter in this study was used only to indicate significant differences regarding the topography characteristics.

**Table 2:** Average Values from Three Measurements (*n* = 3) of the Roughness (Sa), Core Height (Sk), Reduced Peak Height (Spk), Reduced Valley Depth (Svk), and Spk/Coatings Thickness (Spk/*d*) Ratio (*n* = 3)^a^

sample	Sa [μm]	Sk [μm]	Spk [μm]	Svk [μm]	Spk/*d* [% of coating thickness]
Ti	0.21 ± 0.01	0.51 ± 0.01	0.45 ± 0.03	0.18 ± 0.01	—
136_450_n	0.49 ± 0.01	1.42 ± 0.01	0.98 ± 0.04	0.42 ± 0.01	∼18
136_450_sin	0.59 ± 0.07	1.58 ± 0.15	1.37 ± 0.23	0.50 ± 0.04&	∼22
136_450_rec	0.80 ± 0.11&	2.01 ± 0.19&	1.74 ± 0.25&	0.51 ± 0.03&	∼26
136_600_n	0.50 ± 0.01	1.43 ± 0.01	1.04 ± 0.05	0.44 ± 0.02	∼21
136_600_sin	0.60 ± 0.08	1.64 ± 0.22	1.30 ± 0.13	0.45 ± 0.02	∼23
136_600_rec	0.85 ± 0.25	2.25 ± 0.59	1.59 ± 0.39	0.52 ± 0.06	∼20
68_450_n	0.52 ± 0.01	1.52 ± 0.02	0.98 ± 0.01	0.45 ± 0.01	∼17
68_450_sin	0.68 ± 0.13	1.83 ± 0.28	1.43 ± 0.33$	0.50 ± 0.04	∼25
68_450_rec	1.14 ± 0.06$	2.98 ± 0.06$	1.87 ± 0.03$	0.55 ± 0.02$	∼21
68_600_n	0.49 ± 0.01	1.41 ± 0.01	0.98 ± 0.01	0.42 ± 0.01	∼18
68_600_sin	0.57 ± 0.06	1.58 ± 0.15	1.21 ± 0.10	0.46 ± 0.01*	∼22
68_600_rec	0.78 ± 0.13*	2.02 ± 0.29*	1.68 ± 0.26*	0.52 ± 0.03*	∼21

a All data are expressed as means ± SD; &, $, and *: statistically significant difference between the samples in the groups (136_450, 68_450, and 68_600, respectively), as compared to the MAO sample in each group (*p* < 0.05). There is no statistically detected difference between samples in the 136_600 group in our study.

Functional parameters from the Sk group facilitate analyzing the tribological properties of materials.^74,75^ Thus, the following functional parameters of all samples were assessed: core height, reduced peak height, reduced valley depth, and areal material ratios of the scale-limited surface. The results are listed in Table 2. For bone tissue engineering, the Sk parameter may represent the core roughness of the surface over which a load may be distributed after the implantation of biomaterial that contacts the tissues and other bones. Two other parameters, Spk and Svk, are directly related to this feature. The Spk value may represent the nominal height of the coating removed during the running-in operation. Simultaneously, Svk measures the valley’s depth below the core roughness. In the case of implants, it can measure the formation of “pockets”, which will be a convenient place for cell proliferation, enabling a permanent bond of the bone implant with the tissue.^76−79^ It can be noted that ultrasound increased the values of these parameters compared with those obtained for MAO coatings. Particular differences were observed for the features of the coating obtained using unipolar rectangular ultrasound during UMAO (Table 2), which is probably related to the tremendous energy released by these ultrasounds and thus the intensification of the process.^70^ The highest values of these parameters were obtained for sample 68_450_rec and the lowest values for sample 68_600_n. Generally, a relatively low Spk value indicates favorable wear resistance and a high Svk value designates beneficial lubricant retention ability, thereby creating a site suitable for cell proliferation.^74,75^ For biomaterials, it is also essential to know what fraction of the coating would be “worn away” during work in the human body and what would probably remain. Due to this, the authors determined the Spk/coatings thickness (Spk/d) ratio, which could be used as an indicator of the durability of the coating. It can be seen that the ultrasound subtly impaired the durability of the coating, which is perhaps due to the electrolyte fluctuations. Nevertheless, there are no significant differences in the groups, and it can be assumed that the strength of the coatings is at a similar level. The exception is sample 136_450_rec. However, the determined thickness of this sample was characterized by the highest standard deviation, which could affect the disparate Spk/d result. Detailed results, analysis, and discussion of the remaining results obtained from the topography studies [the kurtosis (Sku), the skewness (Ssk), the maximum peak height (Sp), and the maximum valley depth (Sv) as well as surface topographies and the histogram of peak and valley distribution] are presented in Description S1, Figure S3, and Table S1.

Coatings generated on biomaterials should cover the material uniformly so that there would not be weak places, whose durability and corrosion resistance in the environment of body fluids would be inferior.^43−45^ Therefore, it is worth analyzing the isotropy of the surface, as it is influenced by the initial type of surface treatment of the material and the kinematics of the process.^80^ Surface texture is expressed as a percentage. The following degrees of isotropy are assumed:^81^

1. Is < 20%—an anisotropic surface;
2. 20% ≤ Is ≤ 80%—a mixed structure;
3. Is > 80%—an isotropic surface which surface structure is of the P type and the dominant direction of unevenness formation is not observed.

**Figure 5:**
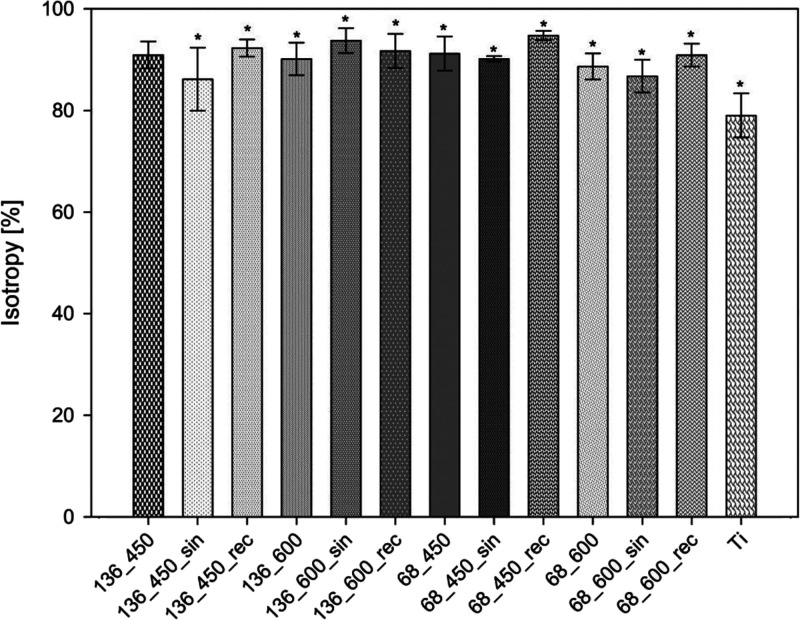
Computed values of surface texture directions. All samples after modification were isotropic. For all analyses *n* = 3; data are expressed as means ± SD; * statistically significant difference as compared to the Ti (*p* < 0.05). There is no statistically detected difference between samples in the groups in our study.

The graphical study of the surface texture directions of all samples is presented in Figure S4, and the computed values of surface texture directions are given in Figure 5. All samples subjected to MAO and UMAO exhibited isotropy (≥86.15 ± 6.21%), while the mechanically polished Ti sample had a mixed structure (Is = 79.02 ± 4.33%). The use of bipolar rectangular ultrasound during the process in each case improved the isotropy, while sinusoidal ultrasound improved the isotropy only when the process was conducted with usage of a current of 136 mA and a time of 600 s. This phenomenon may be related to the previously mentioned ultrasound characteristics. The maximum amplitude ultrasound affects electrolyte fluctuations more and accelerates the MAO process’s kinetics, significantly contributing to surface formation.^71,72^ On the other hand, when improving the surface homogeneity for samples from groups 136_450 and 136_600, when sinusoidal ultrasounds were used, which reached their maximum amplitude only in single moments, the main factor affecting the surface texture was the high current density.^74^ The highest value for isotropy for the sample after modification was found for sample 68_450_rec (94.76 ± 0.92%), whereas the lowest value (86.15 ± 6.21%) is associated with sample 136_450_sin. As the samples were prepared equivalently, the effect of the process on the isotropy of the surface can be observed. Bipolar rectangular ultrasound enhances surface anisotropy, leading to uniform surface structure parameters in all directions.

### 3.4 Coating Thickness

**Figure 6:**
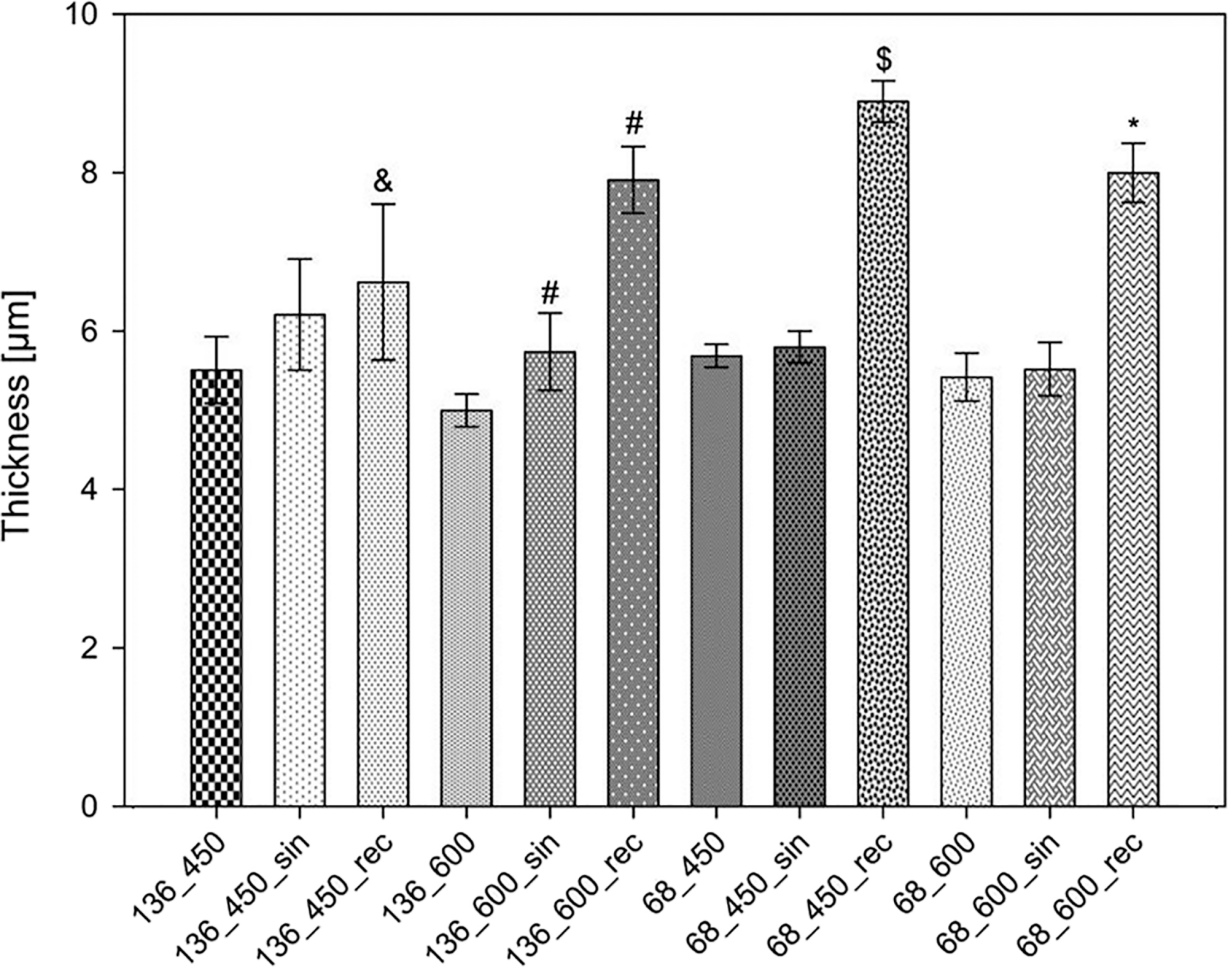
Thickness values of the MAO and UMAO coatings generated under different conditions (*n* = 10). The use of ultrasound during the MAO process increased the thickness of the coatings. All data are expressed as means ± SD; &, #, $, and *—statistically significant difference between the samples in the groups (136_450, 136_600, 68_450, and 68_600, respectively), as compared to the MAO sample in each group (*p* < 0.05).

The thickness of the coatings varies depending on the process parameters used, as listed in Figure 6. In the case of MAO coatings, the values are in the range of 5.0–5.7 μm, and the alteration in current density and time had no significant effect on this parameter. On the other hand, the use of ultrasound during the process significantly increased the thickness of the coatings generated with the same process parameters, since this parameter for UMAO coatings ranges from 5.7 to 8.9 μm. For example, the use of a unipolar rectangular ultrasound wave in the 68_450 group increased the coating thickness from 5.7 ± 0.1 to 8.9 ± 0.3 μm. The use of ultrasound contributed to an increase in the thickness of all coatings, which has also been confirmed in other studies.^66,82^ The use of the unipolar rectangular mode contributed more to the increase in the coating thickness than the sinusoidal mode. According to the characteristics of these modes, unipolar rectangular ultrasound has a maximum amplitude for a more protracted process time than sinusoidal ultrasound. The greater the amplitude, the greater the wave’s energy and, simultaneously, the greater the amount of transported mass.^70^

### 3.5 Adhesion Properties

**Figure 7:**
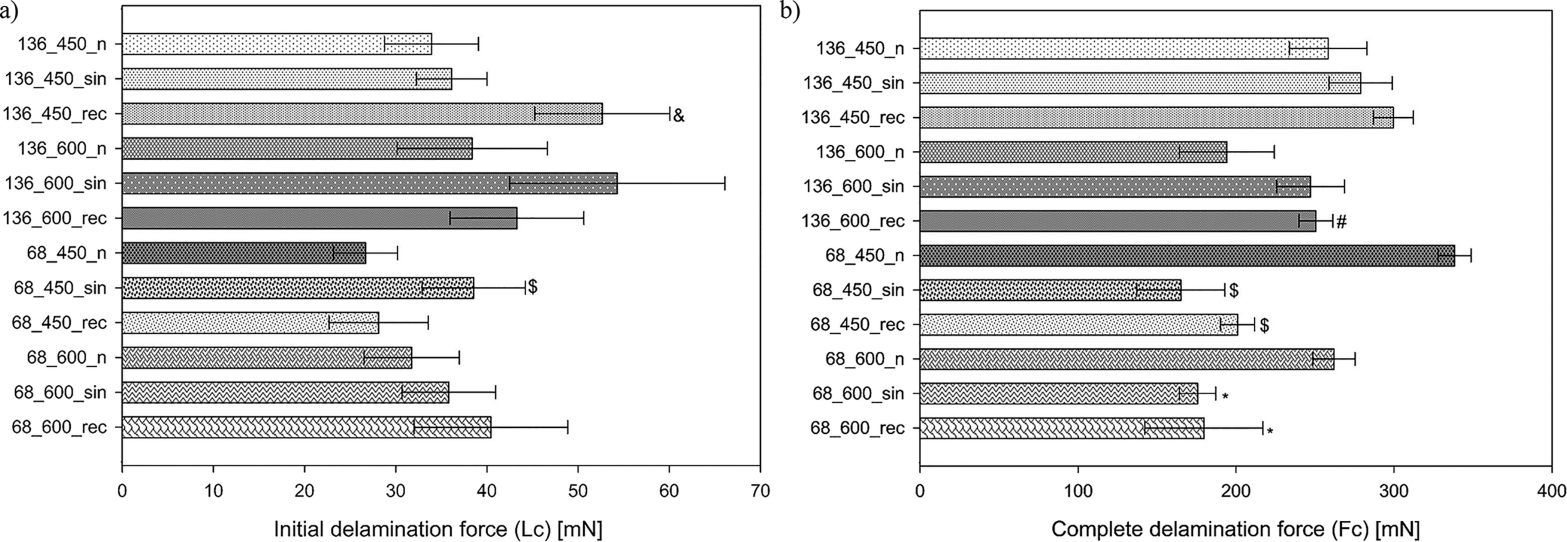
(a) Initial delamination force (*L*_c_) and (b) complete delamination force (*F*_c_) values for the MAO and UMAO coatings produced in different conditions (*n* = 5). The use of ultrasound during the MAO process increased the adhesion of the coatings to the substrate (higher *L*_c_ values for UMAO coatings). All data are expressed as means ± SD; &, #, $, and *—statistically significant difference between the samples in the groups (136_450, 136_600, 68_450, and 68_600, respectively), as compared to the MAO sample in each group (*p* < 0.05).

The initial and complete delamination forces (*L*_c_ and *F*_c,_ respectively) values are presented in Figure 7, and an example of the relation between frictional force and topography with the indicated critical loads for coating is shown in Figure S5. The collected data indicated that using ultrasound during the MAO process increased the initial delamination force. Moreover, increasing the current density and extending the time during the process also increased the adhesion of the coatings to the substrate (higher initial delamination forces), consistent with the results obtained by Yao et al. for Ti-6Al-4 V alloy produced by selective laser melting.^56^ The highest *L*_c_ value was found for sample 136_600_sin and the lowest for sample 68_450_n. In the case of complete delamination force, an increase in the adhesion of UMAO coatings was observed for the process carried out using a current of 136 mA (groups 136_450 and 136_600). In comparison, for the process performed at a current of 68 mA, ultrasonication adversely affected the complete delamination resistance of the coating (group 68_450 and 68_600). Sample 68_450_n remained undamaged for the longest of all analyzed coatings (complete delamination occurred farthest from the scratch path’s beginning, which was ∼670 μm). In contrast, the fastest complete delamination occurred in sample 68_450_sin (complete delamination occurring closest to the beginning of the scratch path - about 350 μm). In general, using ultrasound makes the process more easily activated, which could contribute to more intense discharges on the titanium substrate and increase the external scratch resistance of the coatings.^47^ On the other hand, the possibility of nonlinear effects caused by ultrasound could cause high resistance variability.^77^ MAO is considered to be an effective method of improving coating adhesion to the substrate compared to other widely used surface modification methods; however, the typical brittle behavior is still observable in many studies.^26,56,83^ The tribological properties of MAO coatings (and thus UMAO) can be improved by using an optimal combination of process parameters (e.g., voltage, current, oxidation time, or electrolyte concentration).^26,84^ Unfortunately, the test conditions, material parameters, and randomness of measurements, which are independent factors in scratch tests, prevent reliable comparisons of results between research groups. The results are approximate and should be used mainly for qualitative comparison purposes.^26^

### 3.6 Surface Wettability

**Figure 8:**
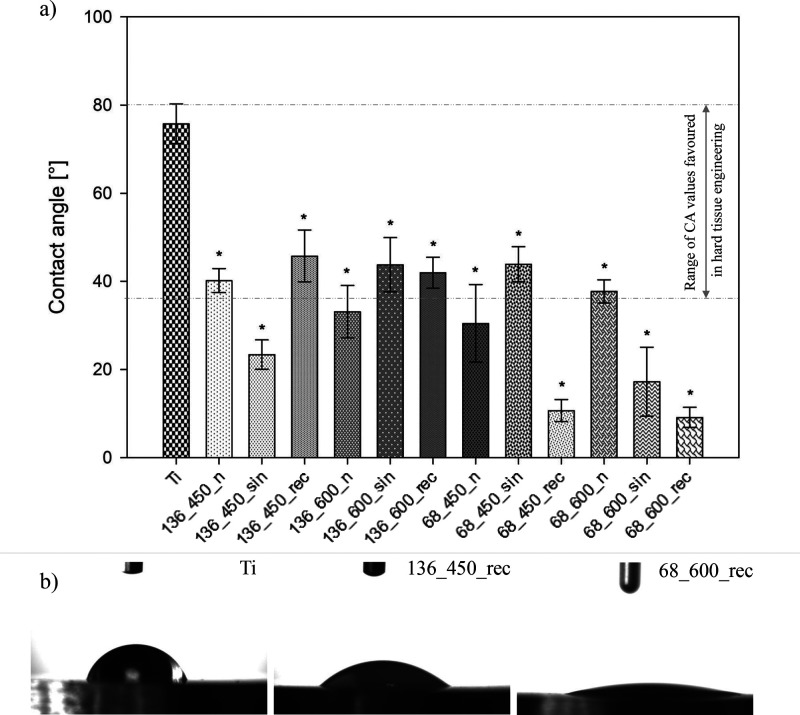
(a) Wettability of specimens determined by water contact angle (*n* = 5; data are expressed as means ± SD; * statistically significant difference as compared to the uncoated Ti (*p* < 0.05)). All samples were hydrophilic in nature. (b) Contact angle for uncoated Ti, 136_450_rec–sample after treatment with the highest contact angle value and 68_600_rec–sample after treatment with the lowest contact angle value.

As surface wettability affects the adhesion of cells, the study of the contact angle was crucial to assess the quality of MAO and UMAO coatings for biomedical applications.^7^Figure 8 presents the wettability measurements of MAO and UMAO coatings on Ti. It was revealed that all specimens were hydrophilic in nature (CA < 90°). The contact angle of the MAO coatings is 30–40°, while UMAO coatings range from 9 to 45°. Sypniewska et al.^83^ generated MAO coatings with contact angles ranging from 32 to 74°. On the other hand, values of the contact angle below 15° were obtained by Li et al.,^85^ who conducted the MAO process on pure titanium in tetraborate electrolyte. In vivo studies on MAO-treated Ti-6Al-4 V suggest that the increase in wettability becomes a significant and objective factor affecting the biological response of MAO coatings. It is considered that the most advisable contact angle value for hard tissue regeneration is 35–80°.^7^ Therefore, the following samples after surface modification were selected for further research: 136_450_n, 136_450_rec, 136_600_sin, 136_600_rec, 68_450_sin, and 68_600_n (Figure 8a). It is stated that the contact angle of MAO coatings increases with the increase of the applied voltage, which can be correlated with, e.g., higher roughness value and/or capillary forces between volcanic pores and contacted distilled water.^26^ However, in this study, there is no relationship between these parameters and, thus, an unequivocal dependence on the influence of ultrasound on the wettability of coatings. However, surface wettability can be strongly affected by other parameters such as morphology, crystallinity, and chemistry.^26^

### 3.7 Biomechanical Properties

Hardness and Young’s modulus are considered critical parameters of ceramic coatings generated on implant material, as their value should be close to that of the replaced bone. In addition, their determination enables the assessment of the antiwear properties of ceramic coatings.^26^Table 3 presents the hardness values of the modified surfaces, their Young’s modulus, *H*/*E* and *H*^3^/*E*^2^ ratio values, and the maximum indentation depth. The highest value of hardness and Young’s modulus was obtained for the sample 68_450_sin (3.53 ± 0.8 and 50.62 ± 4.5 GPa, respectively), while the lowest values of hardness and Young’s modulus were obtained for the 68_600_n sample (1.93 ± 0.8 and 34.65 ± 9.1 GPa, respectively). All MAO and UMAO modifications influence the discussed mechanical features, as Young’s modulus for Ti is 143 ± 23 GPa,^86^ and hardness equals 2.6 ± 0.7 GPa.^86^ Significant is the reduction of Young’s modulus, which should be as close as possible to bone values (10–40 GPa),^87^ and thus the most optimal fit is observed for the 68_600_n sample; however, considering the standard deviation of the measurements, optimal values were also obtained for the samples 136_450_rec, 136_600_sin, and 136_600_rec. In the case of MAO coatings, higher Young’s modulus and hardness values are obtained for materials with a lower porosity level and a dense microstructure.^26^ A similar relationship was obtained in our research, but in the case of samples subjected to the UMAO process using sinusoidal ultrasound, high values of these variables were observed despite the porosity of about ∼25%. Guo et al.^48^ reported that using US during the MAO process on 6063 aluminum substrates increased the hardness of the coatings. We presume that sinusoidal ultrasound, which transmits less energy, promoted cold quenching of the coating. In addition, nonlinear effects caused by ultrasound may have occurred during the formation of the coating on the substrate.^77^

**Table 3:** Nanomechanical Properties of Coatings: Hardness (*H*), Young’s Modulus (*E*), *H*/*E* and *H*^3^/*E*^2^ Ratio Values of the MAO and UMAO Coatings Generated in Different Conditions as Well as Contact Depth and % of Penetration Depth (*n* = 15)^a^

sample	*H* [GPa]	*E* [GPa]	*H*/*E* [—]	*H*^3^/*E*^2^ [GPa]	contact depth [μm]	penetration depth [% of coating thickness]
136_450_n	3.23 ± 0.8	49.05 ± 5.8*	0.064 ± 0.01	0.015 ± 0.008	0.85 ± 0.1#, $	∼15
136_450_rec	2.14 ± 1.1	41.08 ± 12.1*	0.048 ± 0.02	0.007 ± 0.007	1.12 ± 0.3	∼17
136_600_sin	3.28 ± 1.7	44.23 ± 11.5*	0.082 ± 0.08	0.105 ± 0.3	0.99 ± 0.2	∼17
136_600_rec	2.56 ± 0.4	46.89 ± 7.8*	0.055 ± 0.005	0.008 ± 0.002	0.91 ± 0.1#, $	∼16
68_450_sin	3.53 ± 0.8	50.62 ± 4.5*	0.069 ± 0.01	0.018 ± 0.009	0.78 ± 0.1#,$,&	∼13
68_600_n	1.93 ± 0.8	34.65 ± 9.1*	0.053 ± 0.01	0.007 ± 0.006	1.13 ± 0.2	∼21

a All data are expressed as means ± SD; * statistically significant difference compared to the commercially pure titanium Young’s modulus [*E* = 143 ± 23 GPa;^86^ (*p* < 0.05)]; there is no statistically detected difference compared to the commercially pure titanium hardness (*H* = 2.6 ± 0.7 GPa^86^); there is no statistically detected difference between samples for *H*/*E*, *H*^3^/*E*^2^ in our study; #, $, and &—statistically significant difference compared to the contact depth of 68_600_n, 136_450_rec, and 136_600_sin sample, respectively (*p* < 0.05).

The *H*/*E* and *H*^3^/*E*^2^ ratios were also determined based on the nanoindentation results. The highest *H*/*E* value was obtained for the sample 136_600_sin (0.082 ± 0.08) and the lowest for the sample 136_450_rec (0.048 ± 0.02). In the case of the *H*^3^/*E*^2^ parameter, the highest value also applies to the sample 136_600_sin (0.105 ± 0.3) and the lowest (0.007 ± 0.006) to the samples 136_450_rec and 68_600_n. The *H*/*E* ratio refers to elasticity, and the *H*^3^/*E*^2^ ratio is usually used to indicate resistance to plastic deformation in a loaded contact.^88^ The literature indicates that a high *H*/*E* ratio (greater than 0.1) results in high wear resistance.^89^ Hence, the best fracture toughness can be attributed to the UMAO (sinusoidal mode) coating generated at 136 mA and 600 s. On the other hand, there are reports that these indicators do not consider the influence of plasticity on fracture toughness. Although plastic deformation is negligible and elasticity dominates in fracture toughness in ceramic coatings, these parameters can only be used as approximations.^90^ It is also worth noting the relevant values of SD for the nanoindentation results. This is particularly characteristic of porous coatings (and thus MAO and UMAO coatings), due to experimental errors related to the influence of the substrate, the effect of pore densification during indentation, and surface roughness.^26,91^ It is stated that the penetration depth of the indenter should be less than 20% of the coating thickness for thin porous ceramic coatings.^92^ On the other hand, others indicate that the impact of the substrate is incidental if the penetration depth is less than 50% of the coating thickness.^93^ In our research, the penetration depth of the indenter for all coatings meets this requirement and is below 50% of the coating thickness.^92^ Furthermore, almost all samples (except 68_600_n) fulfill the condition that the penetration depth of the indenter should be less than 20% of the coating thickness, which according to the literature should improve the reliability of the result. The methodology for the zero-failure determination of mechanical properties of porous coatings has not yet been well established, but the literature suggests that nanoindentation assays of porous coating can be improved by using a spherical (larger area) tip of the indenter.^26^

### 3.8 Corrosion Properties

The results of corrosion tests are presented in Figure S6 and Table 4. The OCP value on the modified samples increased compared to the uncoated sample (Figure S6), and OCP stabilization occurred at more noble values, indicating that the barrier effect of the coating was not reduced during 1 h of immersion. However, the OCP values differ from the zero current potential (often called the corrosion potential) determined by the software based on Tafel extrapolation. Therefore, our study does not consider the OCP value as a helpful measure of the corrosion tendency. The analysis made on the grounds of the potentiodynamic polarization technique revealed that both coated samples and pure titanium showed an active to passive transition behavior. Moreover, all applied surface modifications have a detrimental effect on the corrosion current density and polarization resistance values, thus on the corrosion rate and protection efficiency of the biomaterial. This relationship is consistent with the results presented by Pawlowski et al.,^63^ who modified titanium surfaces (Ti and Ti-13Zr-13Nb) using different techniques: direct voltage anodic oxidation, electrophoretic deposition, or MAO. The results showed that almost all surface modifications (including MAO), mainly aimed at improving biological properties, negatively affect the corrosion resistance of biomaterials. The observed deterioration in corrosion resistance may be due to coating imperfections (microcracks), possible accelerated degradation of biodegradable deposits in Ringer’s solution during electrochemical tests, or insufficient oxidation of surfaces inside deep pores, followed by potential difference and formation of an electrochemical cell “surface top–pore bottom”.^63^

**Table 4:** Results of Corrosion Assays: Corrosion Current Density (*j*_corr_), Zero Current Potential (*E*_*j*=0_), Polarization Resistance (*R*_pol_), Corrosion Rate (CR), and Protection Efficiency (PE) (*n* = 3)^a^

sample	OCP [mV]	*I*_corr_ [nA/cm^2^]	*E*_*j* = 0_ [mV]	*R*_pol_ [kΩ·cm^2^]	CR [μm/year]	PE [%]
Ti	–255.9 ± 4	33.0 ± 3	–325.6 ± 19	1929.5 ± 217	0.287 ± 0.02	100.0 ± 9
136_450_n	190.6 ± 1*	247.6 ± 4*	–331.7 ± 16	329.1 ± 21*	2.151 ± 0.03*	–649.4 ± 10*
136_450_rec	141.1 ± 4*	252.1 ± 25*	–307.8 ± 9	273.2 ± 10*	2.189 ± 0.21*	–662.9 ± 64*
136_600_sin	151.5 ± 1*	266.1 ± 11*	–319.0 ± 7	264.6 ± 20*	2.311 ± 0.10*	–705.4 ± 30*
136_600_rec	97 ± 1*	250.4 ± 27*	–306.6 ± 1	290.2 ± 14*	2.175 ± 0.23*	–657.9 ± 71*
68_450_sin	185.6 ± 3*	280.5 ± 24*	–333.9 ± 1	274.3 ± 11*	2.437 ± 0.21*	–749.0 ± 64*
68_600_n	132.0 ± 3*	245.7 ± 25*	–341.3 ± 1	298.7 ± 16*	2.134 ± 0.22*	–643.6 ± 65*

a All data are expressed as means ± SD; * statistically significant difference compared to the Ti sample (*p* < 0.05).

In the case of samples from the same group, no significant impact of using ultrasound or changing the ultrasonic mode on the corrosion resistance was observed. However, a dependence was noticed: the greater the porosity of the coating, the worse the corrosion resistance. In our study, the best corrosion resistance for the modified samples was obtained for 68_600_n, and the worst corrosion resistance was obtained for 68_450_sin. However, the corrosion current densities are still very low, i.e., values of the order of nA/cm^2^ (range 245.7–280.5 nA/cm^2^). For example, Bordbar-Khiabani et al.^94^ obtained reduced graphene oxide/titanium dioxide composite coatings on pure titanium using the MAO process, and the corrosion current density for the modified samples ranged from 6.8 to 485 nA/cm^2^. Furthermore, the corrosion rate of the modified MAO and UMAO samples (Table 4) is in the range of 2.134–2.437 μm/year and well below 130 μm/year, which is the maximum corrosion rate commonly accepted for biomaterial design and application.^95^ Thus, our modifications can be successfully used in bone tissue engineering. Nevertheless, perhaps the applied high voltage had a decisive influence on the corrosion resistance of the modified substrate. Studies show that lower voltage during modification is probably more promising in generating anticorrosion coatings.^63^

### 3.9 Cytocompatibility

**Figure 9:**
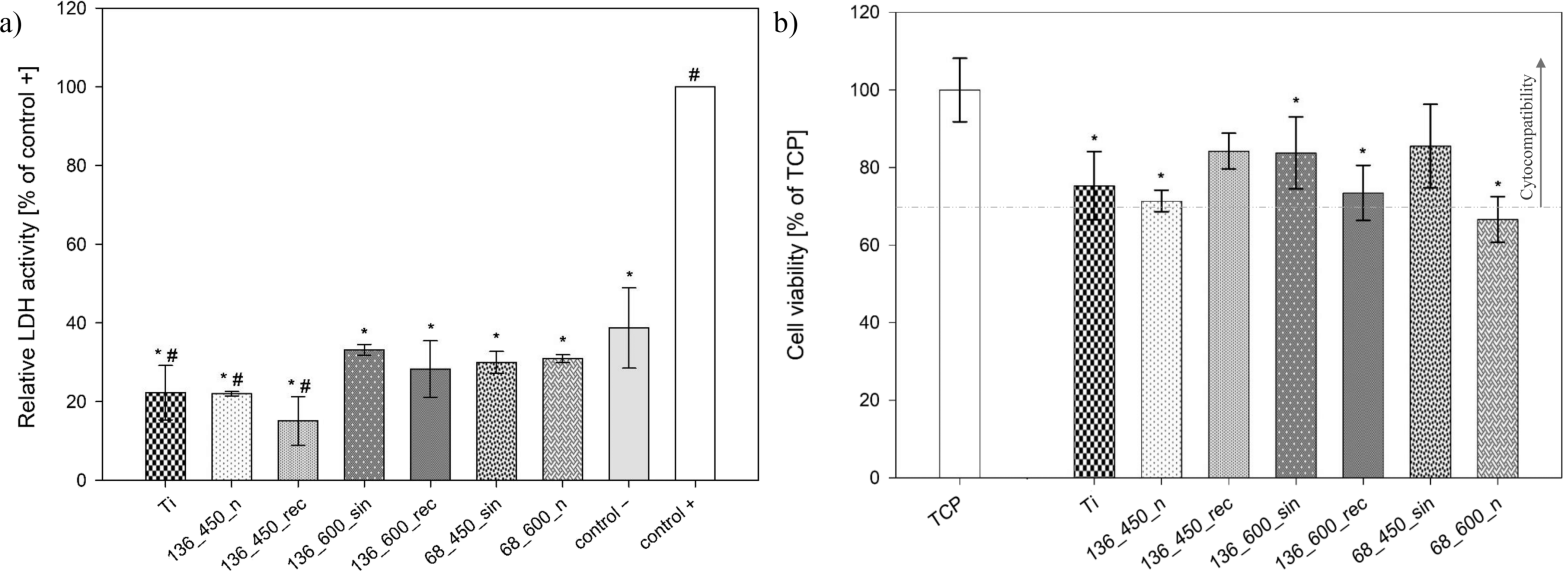
(a) LDH release after in vitro 72 h exposure of hFOB to tested specimens (*n* = 4; data are expressed as means ± SD; * statistically significant difference as compared to the control (+) (*p* < 0.05); # statistically significant difference as compared to the control (−) (*p* < 0.05). The control group (−) represents the negative control for cell death, while the control group (+), in which Triton X-100 was added, represents the positive control for cell death. (b) fHOB viability on tested specimens after 3-day culture. Results are expressed as a percentage of cell viability compared to the cell viability on the TCP (*n* = 4; data are expressed as the mean ± SD; * statistically significant difference as compared to TCP (*p* < 0.05). Except for sample 68_600, the other coatings are cytocompatible.

The cytocompatibility of the selected MAO and UMAO coatings was tested with the hFOB 1.19 osteoblast cell line and direct method by seeding the cells onto the coating.^96^ The first method of determining cytotoxicity was based on measuring the activity of cytoplasmic enzymes released by damaged cells. The amount of LDH released is directly proportional to the number of cells undergoing apoptosis, necrosis, or other cellular damage.^97^ As shown in Figure 9a, the LDH release was slightly lower for the tested samples than the control (−), reflecting the basal level of cell death in such cell culture on a tissue culture plate.^97^ The LDH release for the modified samples ranges from ∼15 to ∼33%, while that for the CP-Ti sample was ∼22%. Hence, the surface modification did not lead to cell damage since the LDH release activity was relatively similar to that of the CP-Ti sample.

Application of the modification and process parameters affected the viability of hFOB 1.19 after 72 h of culture (Figure 9b). Generally, samples after UMAO treatment were characterized by increased cell viability compared to samples after the MAO process. Except for sample 68_600_n, the others can be classified as cytocompatible because the cell viability in response to the tested samples was above 70%.^98^ For samples 136_450_rec, 136_600_sin, and 68_450_sin, cell viability was higher than that for uncoated titanium. It is known that osteoblast viability depends on many variables, such as the coatings’ chemical composition, topography, and wettability.^7^ For example, Tsai et al.^99^ showed that MAO coatings on Ti-x*Zr films deposited using a cathodic arc deposition showed higher osteosarcoma cell viability (MG-63) with higher Zr content (x*). On the other hand, the cell viability of human skin fibroblast cells cultured on Ti-0.7Zr film was lower than for the sample without the addition of zirconium. Cytotoxicity studies performed by Xu et al.^100^ on pure titanium showed that MAO coatings obtained in phosphate sodium solution (10g/L) were characterized by better cytocompatibility than pure titanium (cell proliferation of neonatal rat’s calvaria cells was higher for the modified sample). On the other hand, adding silicon to the electrolyte decreased cell viability in vitro studies despite being considered a bioactive material that can promote adhesion, proliferation, and differentiation of osteoblast-like cells.^100^ Our work confirmed that hFOB cells’ viability was enhanced compared to uncoated Ti. We also found decreased cell viability for coatings with lower contact angles (∼37–41°) and the opposite effect for samples with greater porosity (in the range of 23–24%). Moreover, it can be concluded that when the Ssk value is relatively high (in the range of 0.90–1.02), hFOB cell viability is close to standard conditions [100%, tissue culture plate (TCP)], while its reduction worsens the cellular response. Higher Ssk values could maximize the contact surface of the cells with the coating.^77^

### 3.10 Optimal MAO/UMAO Modification for Pure Ti

The MAO method is a relatively novel method in which the coating’s formation mechanism has not yet been studied in detail.^7^ Due to the many variable parameters of the process (time, voltage, current, electrolyte composition, etc.), as well as the possibility of modifying this process by using other techniques (e.g., ultrasound), finding the optimal combinations of these variables, which would enable the development of coatings for biomedical applications, is incredibly intricate. Since, in our work, the coatings were designed to be used in implantology, we created them based on calcium phosphates—which allowed us to impart bioactivity to the titanium substrate and ensured high biocompatibility as a reflection of natural bone. The selection of an appropriate CaP coating for biometals, like titanium, is related to the proper assessment of their properties, such as morphology, thickness, crystallinity, roughness, chemical composition, corrosion resistance, wettability, surface energy, mechanical, tribological and adhesive properties, as well as in vitro and in vivo assays.^7^ In this study, several experiments facilitated the selection of the most optimal MAO/UMAO process variables, and their summary concerning the requirements for modern biofunctional coatings is included in Table 5.

**Table 5:** Requirements for Modern Biofunctional Coatings Based on CaP Dedicated for Biometallic Implants Compared to the Obtained Results for the Developed Coatings with Various MAO/UMAO Process Parameters^a^

sample	requirements						
	microstructure with favorable porosity^68^	chemical composition (Ca/P ratio)^26^	isotropy (above 80%)^43−45,80^	wettability (contact angle in the range 35–80°)^7^	mechanical properties (bone-like stiffness)^26^	corrosion resistance (ensuring the stability of the coating)^95^	cytocompatibility (osteoblast viability above 70%)^98^
136_450_n	S	S	S	S	U	S	S
136_450_sin	S	S	S	U	—	—	—
**136_450_rec**	**S**	**S+**	**S+**	**S**	**S+**	**S**	**S+**
136_600_n	S	S	S	U	—	—	—
136_600_sin	S	S	S+	S	S	S	S+
136_600_rec	S	S	S	S	S	S	S
68_450_n	S	S	S	U	—	—	—
68_450_sin	S	S	S	S	U	S	S+
68_450_rec	S	S+	S+	U	—	—	—
68_600_n	S	S	S	S	S+	S	U
68_600_sin	S	S	S	U	—	—	—
68_600_rec	S	S+	S	U	—	—	—

a S: Meets the requirement (satisfied properties); S+: the most favorable result for the tested property (max. 3 chosen samples; Ca/P ratio: the closer the value is to 1.67; isotropy: the highest; mechanical properties: the closer the value of Young’s modulus to 10–40 GPa; cytocompatibility: the higher the cell viability value); U: does not meet the requirement (underperforming properties).

The studies show that using the selected variables enabled the generation of porous coatings with an isotropic structure, in which the chemical composition is similar to hydroxyapatite’s natural bone-building material. Their topography (mainly the discussed roughness and skewness), thickness, and adhesive properties strongly depended on the process parameters. However, the exact requirements for the “ideal” roughness value (and other topography parameters that may affect cell adhesion, proliferation, and differentiation), thickness, and tribological properties for biomedical applications have not yet been precisely defined.^7^ Therefore, these parameters are deeply discussed in the sections above. Briefly, based on literature analysis, we assumed that (1) the CaP ratio should be above 1.2 and close to 1.67,^26^ (2) the contact angle value should be between 35 and 80°,^7^ (3) the isotropy should be above 80%,^81^ (4) skewness should be above 0.90, (5) the corrosion rate should be below 130 μm/year,^95^ (6) Young’s modulus should be 10–40 GPa,^26^ and finally, (7) the cell viability greater than pure titanium and close to TCP (100%).^98^ Therefore, we may confirm that all adopted requirements were met by samples in which UMAO modification was carried out at a current of 136 mA, time 450 s, and unipolar rectangular US, and modification performed at a current of 136 mA, time 600 s, and sinusoidal or unipolar rectangular US. Further, we believe the most optimal coating for pure titanium dedicated to biomedical applications is generated on 136_450_rec, as shown in Table 5.

### 3.11 Cell Adhesion and Proliferation

**Figure 10:**
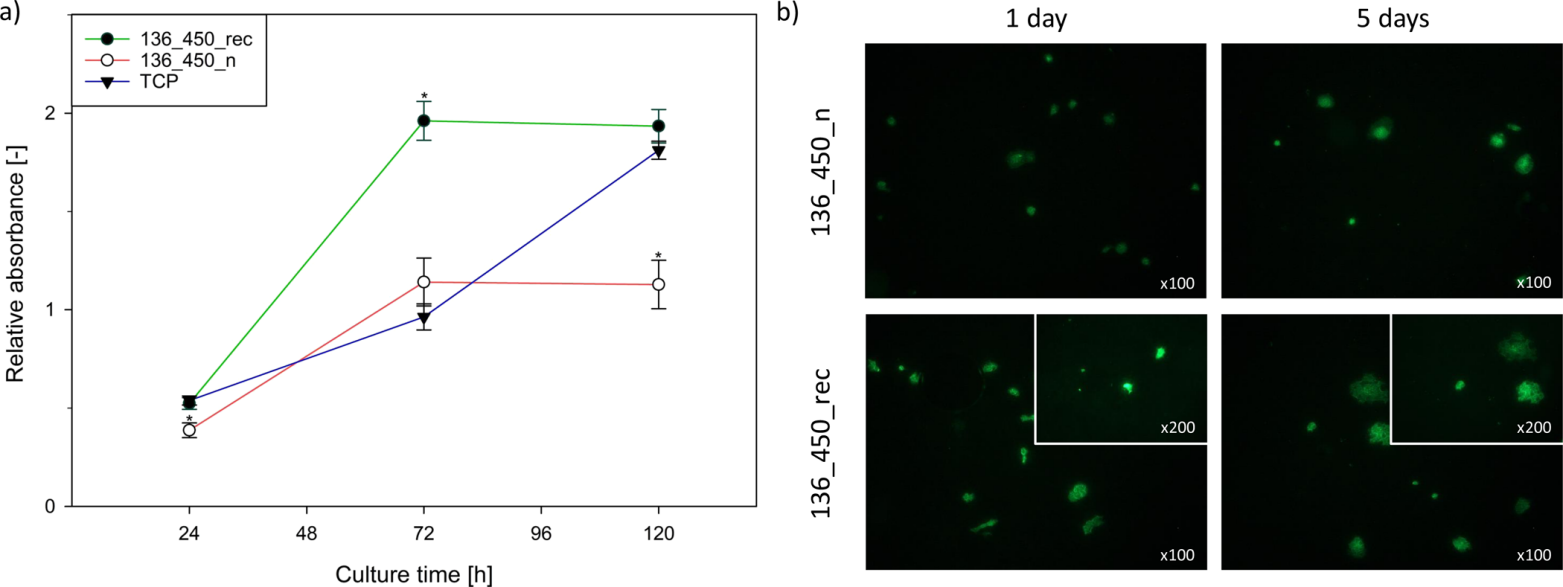
(a) Relative absorbance (*n* = 4) as a function of cell proliferation after 1, 3, and 5 days of in vitro incubation. On the fifth day, absorbance was 1.13 ± 0.14 for the 136_450_n sample and 1.93 ± 0.08 for the 136_450_rec sample; * statistically significant difference as compared to the TCP (*p* < 0.05). (b) Fluorescence microscope images of hFOB 1.19 cells adhered to the surface of 136_450_n and 136_450_rec samples after 1 and 5 days of incubation.

The capability of the cells to adhere to the implant surface determines their posterior proliferation and differentiation, and consequently, the formation of the permanent tissue–biomaterial interface.^24^ As the research examines the influence of ultrasound on the characteristics of coatings obtained in micro-arc oxidation process concerning the biomedical application, the adhesion and proliferation of hFOB 1.19 osteoblast cells on the surfaces of 136_450_n (MAO) and 136_450_rec (UMAO; noted as optimal coating) samples were checked at different time points (24, 72, and 120 h). The MTT results (Figure 10a) revealed that after 1 day of culture, cell proliferation on different surfaces was comparable to the proliferation in standard conditions. After 3 days, a significant increase in absorbance (almost 4-fold compared to the first day) was observed for the 136_450_rec sample, while the absorbance for TCP and the 136_450_n sample increased approximately 2-fold). The results obtained after 5 days of culture showed that cell proliferation was inhibited on the surfaces of the modified samples without causing their death (absorbance for the modified samples was similar to the ones on days 3 and 5). The use of ultrasound during the MAO process resulted in the formation of coatings on which cell proliferation was approximately 2 times greater (after 3 and 5 days). Fluorescence microscopy images (Figure 10b) confirmed that the line of osteoblast-like cells after 1 and 5 days of incubation attached the surface of the MAO and UMAO coatings. In the case of sample 136_450_n, the number of cells on the appropriate days of incubation was lower than that in the case of sample 136_450_rec. The cells are distributed more evenly on the UMAO coating than on the MAO coating, which may be related to the greater porosity and regularity of the microstructure of the UMAO coating.^101^ The cells are characterized by different morphologies depending on the incubation time. After 1 day, cells show a relatively round structure, indicating short filopodia around the cellular body.^24^ However, after 5 days of incubation, the cells are effused, which may indicate growth and elongation of the filopodia and lamellipodia around the cellular body.^102^ A distinct change in shape was observed in the case of sample 136_450_rec, where after 5 days of incubation, the cells were characterized by a more spread-out morphology, which may cause them to be strongly connected to the sample surface.^24^ Our findings are consistent with those of other authors^24,101,103^ and indicate good cytocompatibility of porous MAO coatings, and our research shows that item UMAO coatings.

## 4 Conclusions

In this study, the surface of pure titanium (grade 2) was successfully modified by the microarc oxidation and ultrasound microarc oxidation processes using a solution containing Ca and P compounds. The influence of the types of ultrasound mode, sinusoidal wave, and unipolar rectangular wave on the characteristics of the coatings was investigated. Moreover, the processes were performed in different process parameters, at various times and current values, to verify the schematics of ultrasound effects. The results indicate that the various MAO process parameters, as well as the use of different types of ultrasound, significantly affect the chemical composition, topography, wettability, and mechanical and adhesive properties of the coatings. We have found that using ultrasound for the MAO process significantly increases coating thickness, improves porosity and pore size, contributes to high isotropy, and also accelerates their roughness and skewness. In addition, ultrasound application increases the level of calcium incorporation, approaching the appropriate Ca/P ratio. Further, all obtained coatings were hydrophilic, showed high porosity, were characterized by diverse microstructure, and had a corrosion rate accepted for biomaterials. We also confirmed their suitable cytocompatibility except for sample 68_600_n. Our observations showed that coatings with a porosity close to ∼23%, a contact angle of ∼45°, and a skewness in the range of 0.90–1.02 contribute to the greatest adhesion and proliferation of osteoblasts. Finally, our research concluded that the optimal conditions for the MAO process are a current of 136 mA, time 450 s, and unipolar rectangular US. Thus, the use of the proposed modification on titanium implants will contribute to its better biofunctionality in applications such as (i) partial joint resurfacing of the knee or hip, (ii) craniofacial reconstruction, or (iii) spinal interbody fusion.

## Supporting Information Available

Procedure for determining corrosion rate (CR) and protection efficiency (PE) parameters; procedure for determining cytocompatibility; analysis and discussion of the results obtained in topography studies; scheme of the sinusoidal and unipolar rectangular waves–two modes used during the UMAO process; surface morphology of (a) 136_600_n, (b) 136_600_sin, (c) 136_600_rec, (d) 68_600_n, (e) 68_600_sin, and (f) 68_600_rec coatings; surface topographies and the histogram of peak and valley distribution of (a) 136_450_n, (b) 136_450_sin, (c) 136_450_rec, (d) 136_600_n, (e) 136_600_sin, (f) 136_600_rec, (g) 68_450_n, (h) 68_450_sin, (i) 68_450_rec, (j) 68_600_n, (k) 68_600_sin, (l) 68_600_rec coatings, and (m) mechanically polished Ti; the graphical study of surface texture directions in Cartesian graphs for samples; the scratch path and the relationship of frictional force, penetration depth, and scratch distance on normal force with the indicated critical loads (Lc and Fc) of the 136_450_rec sample; (a) open-circuit potential vs time and (b) potentiodynamic polarization curves recorded for uncoated Ti and Ti after modifications; the average values from three measurements (*n* = 3) of the kurtosis (Sku), the skewness (Ssk), the maximum peak height (Sp), and the maximum valley depth (Sv) (PDF)

## Abbreviations

ANOVA: analysis of variance
β-GPNa: β-glycerophosphate disodium salt pentahydrate
CA: contact angle
CAH: calcium acetate hydrate
CR: corrosion rate
*d*: thickness;
*E*: Young’s modulus
EDS: X-ray energy-dispersive spectrometer
*E*_*j*=0_: zero current potential
*F*_c_: complete delamination force
*H*: hardness
hFOB: human fetal osteoblast cell
Is: isotropy
*j*_corr_: corrosion current density
*L*_c_: initial delamination force
LDH: lactate dehydrogenase
MAO: microarc oxidation
MTT: 3-(4,5-Dimethylthiazol-2-yl)-2,5-diphenyltetrazolium bromide assay
NADH: 1,4-dihydronicotinamide adenine dinucleotide
OCP: open circuit potential
PBS: phosphate-buffered saline
PE: protection efficiency
PFA: paraformaldehyde
*R*_pol_: polarization resistance
Sa: roughness
SCE: saturated calomel electrode
SD: standard deviations
SED: secondary electron detector
SEM: scanning electron microscope
Sk: average core height
Sku: kurtosis
Sp: maximum peak height
Spk: reduced peak height
Ssk: skewness
Sv: maximum valley depth
Svk: reduced valley depth
TCP: tissue culture plate
UMAO: ultrasound microarc oxidation
US: ultrasound
