# Supplementary material for: Influence of Ultrasound on the Characteristics of CaP Coatings Generated Via the Micro-arc Oxidation Process in Relation to Biomedical Engineering
Source: ACS Biomater Sci Eng. 2024 Mar 19;10(4):2100–15. doi: 10.1021/acsbiomaterials.3c01433 (PMC11005015; doi:10.1021/acsbiomaterials.3c01433)
Supplement: Supplementary file 1 — ab3c01433_si_001.pdf [file ab3c01433_si_001.pdf]

Supporting Information for

The Influence of Ultrasound on the Characteristics of CaP Coatings Generated via Micro-Arc Oxidation Process in Relation to Biomedical Engineering

Balbina Makurat-Kasprolewicz [1,*], Marcin Wekwejt [2], Anna Ronowska [3], Grzegorz Gajowiec [1], Marlena Grodzicka [4], Stefan Dzionk [5], Agnieszka Ossowska [1]

[1] Department of Materials Science and Technology, Gdansk University of Technology, 80-233 Gdańsk, Poland
[2] Department of Biomaterials Technology, Gdansk University of Technology, 80-233 Gdańsk, Poland
[3] Department of Laboratory Medicine, Medical University of Gdańsk, 80-210 Gdańsk, Poland
[4] Faculty of Chemistry, Nicolaus Copernicus University in Toruń, 87-100 Toruń, Poland
[5] Department of Manufacturing and Production Engineering, Gdansk University of Technology, 80-233 Gdańsk, Poland
* Correspondence: balbina.makurat-kasprolewicz@pg.edu.pl; tel.: +48-58-347-14-65

Number of protocols: 2
Number of descriptions: 1
Number of figures: 6
Number of tables: 1
Number of references: 9

Supporting Protocols

Protocol S1: Procedure for determining corrosion rate (CR) and protection efficiency (PE) parameters

In accordance with an ASTM G102-89 standard [1], the following equation was used to calculate the CR for all samples:

$$\mathrm{CR}\ [\mu\mathrm{m/yr}] = \mathrm{K}\frac{\mathrm{j_{corr}}}{\rho}\mathrm{EW} \times 1000, \quad (1)$$

where K – constant equals 3.27, EW – equivalent weight, which for Ti is 11.98 and is considered dimensionless in the calculations, ρ – density of Ti equals 4.51 g/cm^3, j_{corr} – corrosion current density [mA/cm^2].
Whereas the following formula was used to determine PE [2]:

$$\mathrm{PE}\ [\%] = \frac{\mathrm{j_{corr_{Ti}}} - \mathrm{j_{corr}}}{\mathrm{j_{corr_{Ti}}}} \times 100, \quad (2)$$

where $j_{corr_{Ti}}$ – the corrosion current density value for uncoated Ti, j_{corr} – the corrosion current density values for coated samples.

Protocol S2: Procedure for determining cytocompatibility

LDH assay:

The culture medium after experiments was used for the lactate dehydrogenase (LDH, fractional (S)-lactate: NAD+ oxidoreductase) release assay. Lactate dehydrogenase is an enzyme located in the cytoplasm [3], and its release into the medium is increased during the death of necrotic cells; therefore, it is used as a marker [4]. LDH activity was determined by measuring NADH oxidation at 340 nm on a spectrophotometer (Ultrospec 3100, Amersham-Pharmacia-Biotech, Cambridge, UK). Control (+)

was the cell cultured on a tissue culture plate (TCP) treated with 0.2% Triton™ X-100, which showed the maximal LDH release (100%).

MTT assay:

The MTT (3-(4,5-dimethylthiazol-2-yl)-2,5-diphenyltetrazolium bromide) assay evaluated the activity of mitochondrial dehydrogenases. This concerns the conversion of yellow 3-(4,5-dimethylthiazol-2-yl)-2,5-diphenyltetrazolium bromide to a blue formazan product [3]. This reaction occurs only in living cells. After 72 h of experiments, fresh culture medium with 0.60 mmol/L MTT was added to the holes, and the culture was pursued the posterior 4 h. A spectrophotometric measurement at 570 nm assessed formazan formation with a microplate reader (VICTOR 1420 Multilabel Counter, PerkinElmer, Warsaw, Poland). The results were expressed as a % change in the live cell number compared to cells cultured on TCP (assumed as 100%).

Supporting Descriptions

Description S1: Analysis and discussion of the results obtained in topography studies

The fourth-order standardized moment of the height distribution is kurtosis, which measures the distribution's flattening of the surface compared to the normal distribution [5]. Generally, for almost all samples (except the 68_450_rec sample) Sku values are above 3.0 value (Table S1), and it can be assumed that the distribution is more concentrated than the normal distribution. As depicted in Figure S3 these kurtosis are visualized as a thin "bell" and are recognized as leptokurtic [6]. For sample 68_450_rec (Figure S3i), the distinctive "thickening" of the tails is observable, and this kurtosis is recognized as platykurtic [6]. For this sample, the Sku value is 2.36±0.32, meaning the distribution is less concentrated. In general, the distribution of the peak and valley is the most symmetrical for samples after the MAO process (Figures S3a, S3d, S3g, S3j) and the most concentrated area is observed for about ~0 µm. This means that the surface is characterized by a similar number of peaks and valleys. For samples modified with the UMAO process performed using sinusoidal ultrasounds, an increase in the number of valleys was observed contrary to the distribution of the number of peaks inasmuch as the peak of the histogram spectrum is observed in the range –0.4–0.2 µm (Figures S3b, S3e, S3h, S3k). For samples modified during the UMAO process carried out with the use of bipolar rectangular ultrasound, an increase in the distribution of the number of peaks in comparison with the distribution of the number of valleys can be seen, and the most concentrated area occurs in the range –1–0 µm (Figures S3c, S3f, S3i, S3l), which strongly depends on the process parameters. Third-order standardized moments of the height distribution is skewness, which enables the assessment of the asymmetry of the height distribution function [7]. Ssk shows the relationship between the occurrence of peaks and valleys. Positive Ssk values for all samples implicate that the surface peaks are dominant in the surface structure. In addition, low values of this parameter indicate that the probability of crack nucleation is low [8]. The most robust asymmetry is observed for sample 136_450_rec, and the weakest can be attributed to sample 68_450_rec. It was noticed that increasing the time of the MAO process increases the asymmetry, and decreasing the current decreases it. In the case of UMAO modifications, no trends were observed. These changes probably occur due to non-linear effects caused by ultrasound that occur during the formation of the coating on the substrate [9]. Other parameters describing the surface topography in biomaterials are Sp and Sv. They refer to the maximum values, i.e. inform about the maximum value of the peak and valley, respectively. It was noted that using ultrasound during the MAO process resulted in higher Sp and Sv values. These observations are consistent with SEM images of the microstructure, which indicated the presence of volcano-like pores for coatings generated during the UMAO process. Taking into consideration the standard deviations of these parameters, it can be

concluded that the obtained coatings are relatively homogeneous and their structures are easily reproducible.

Supporting Figures

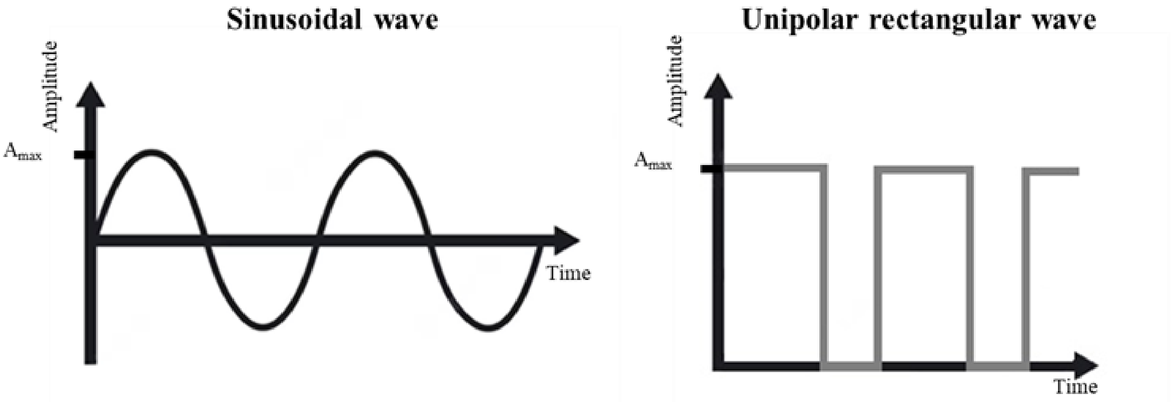

Figure S1. Scheme of the sinusoidal and unipolar rectangular waves – two modes used during the UMAO process.

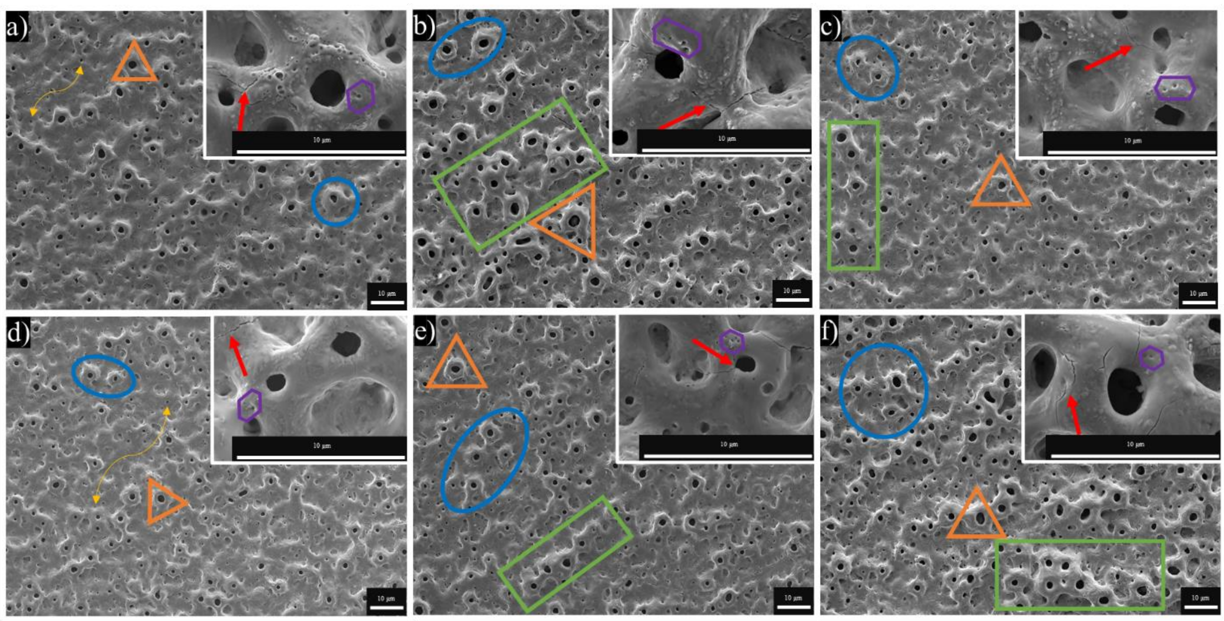

Figure S2. Surface morphology of a) 136_600_n, b) 136_600_sin, c) 136_600_rec, d) 68_600_n, e) 68_600_sin, f) 68_600_rec coatings. Porous coatings were obtained, and their structure depended on the parameters used. Legend: green quadrangle – net formation; blue oval – volcano-like pores; red arrow – micro-crack; yellow double arrow – plane supermicron pores; violet hexagon – submicron pores; orange triangle – micropores.

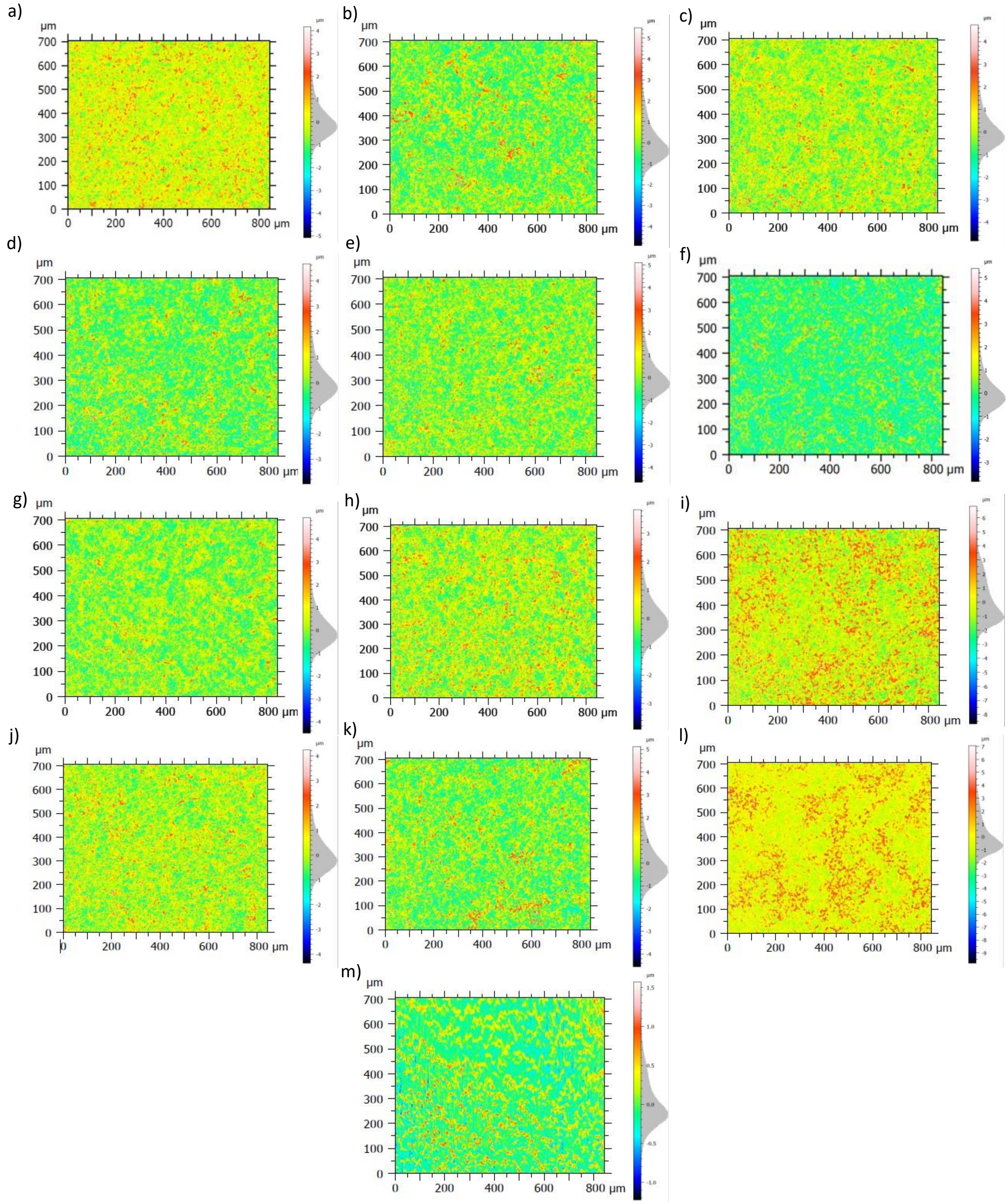

Figure S3. Surface topographies and the histogram of peak and valley distribution of a) 136_450_n, b) 136_450_sin, c) 136_450_rec, d) 136_600_n, e) 136_600_sin, f) 136_600_rec, g) 68_450_n, h) 68_450_sin, i) 68_450_rec, j) 68_600_n, k) 68_600_sin, l) 68_600_rec coatings and m) mechanically polished Ti. For sample 68_450_rec the distinctive “thickening” of the tails in the histogram is observable, and this kurtosis is recognized as platykurtic, while the other histograms are visualized as a thin "bell" and are recognized as leptokurtic.

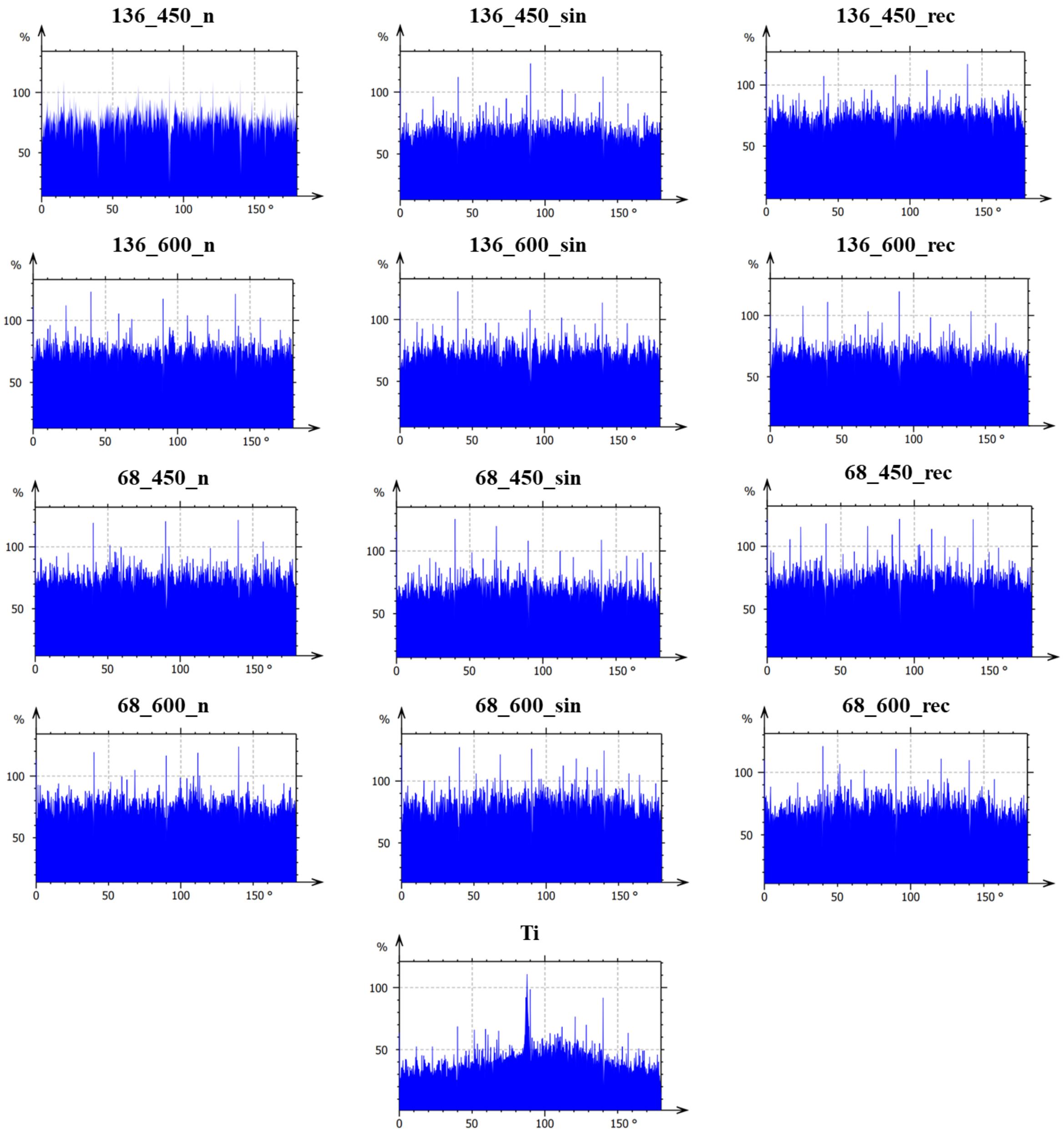

Figure S4. The graphical study of surface texture directions in Cartesian graphs for samples.

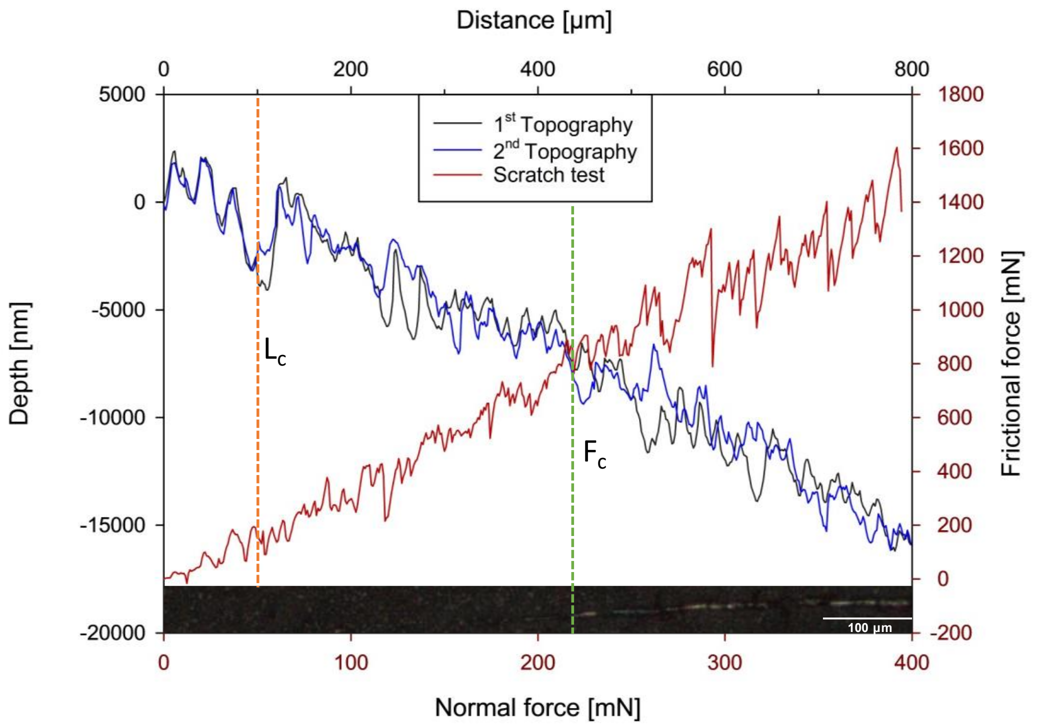

Figure S5. The scratch path and the relationship of frictional force, penetration depth, and scratch distance on normal force with the indicated critical loads (L_c and F_c) of the 136_450_rec sample. L_c was identified by first differences in topography before and after the scratch test. F_c was measured by observing the scratch path by optical microscopy and using the relationship of friction and normal forces. The presented results are representative for five analyses.

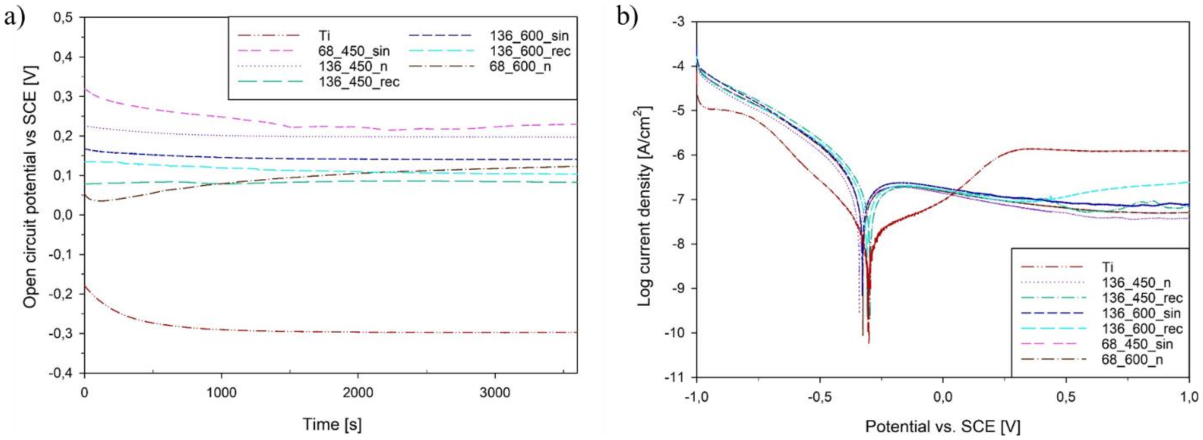

Figure S6. a) Open-circuit potential vs. time and (b) potentiodynamic polarization curves recorded for uncoated Ti and Ti after modifications. The research was performed in Ringer's solution at a scan rate of 0.3 mV/s at 37 °C. Surface modification deteriorated the corrosion resistance of the biomaterial. The presented results are representative of three analyses of each surface treatment specimen.

Supporting Tables

Table S1. The average values from three measurements (n=3) of the kurtosis (Sku), the skewness (Ssk), the maximum peak height (Sp), and the maximum valley depth (Sv).

Sample	Sku [—]	Ssk [—]	Sp [µm]	Sv [µm]
Ti	3.51±0.09	0.78±0.03	1.83±0.47	1.35±0.24
136_450_n	4.35±0.17	0.81±0.04	4.14±0.19	3.46±0.60
136_450_sin	4.76±0.05	1.00±0.04 &	4.66±0.17	3.52±0.60
136_450_rec	4.17±0.33	1.02±0.08 &	6.43±1.16 &	4.28±0.48
136_600_n	4.40±0.10	0.84±0.04	4.63±0.17	3.27±0.41
136_600_sin	4.51±0.75	0.97±0.15	5.17±0.35	4.04±0.48
136_600_rec	3.55±0.94	0.78±0.16	5.20±0.44	5.36±2.11
68_450_n	4.37±0.43	0.76±0.07	5.13±0.97	3.68±0.65
68_450_sin	4.26±0.20	0.90±0.12	6.73±3.48	3.73±0.26
68_450_rec	2.36±0.32 $	0.61±0.06	5.38±0.15	4.44±0.17
68_600_n	4.19±0.010	0.79±0.02	4.03±0.17	3.46±0.60
68_600_sin	4.46±0.11	0.93±0.01 *	4.92±0.38	3.48±0.14
68_600_rec	4.19±0.55	0.97±0.09 *	7.03±1.68 *	4.75±0.70

All data are expressed as means ± SD; &, $, * - statistically significant difference between the samples in the groups (136_450, 68_450 and 68_600, respectively), as compared to the MAO sample in each group ($p < 0.05$). There is no statistically detected difference between samples in the 136_600 group in our study.

References

1. International A. ASTM Standard G102-89, "Standard Practice for Calculation of Corrosion Rates and Related Information from Electrochemical Measurements". ASTM International: West Conshohocken; 2015.
2. Shabani-Nooshabadi M, Karimian-Taheri F. Electrosynthesis of a polyaniline/zeolite nanocomposite coating on copper in a three-step process and the effect of current density on its corrosion protection performance. Rsc Advances 2015;5(117):96601-96610, doi:10.1039/c5ra14333k
3. Bartmanski M, Rosciszewska M, Wekwejt M, et al. Properties of New Composite Materials Based on Hydroxyapatite Ceramic and Cross-Linked Gelatin for Biomedical Applications. International Journal of Molecular Sciences 2022;23(16), doi:10.3390/ijms23169083
4. Kendig DM, Tarloff JB. Inactivation of lactate dehydrogenase by several chemicals: Implications for in vitro toxicology studies. Toxicology in Vitro 2007;21(1):125-132, doi:10.1016/j.tiv.2006.08.004
5. Trzepiecinski T, Szpunar M, Dzierwa A, et al. Investigation of Surface Roughness in Incremental Sheet Forming of Conical Drawpieces from Pure Titanium Sheets. Materials 2022;15(12), doi:10.3390/ma15124278
6. Kallner A. Formulas. Laboratory Statistics: Methods in Chemistry and Health Sciences, 2nd Edition 2018;1-140, doi:10.1016/b978-0-12-814348-3.00001-0
7. Brys G, Hubert M, Struyf A. A robust measure of skewness. Journal of Computational and Graphical Statistics 2004;13(4):996-1017, doi:10.1198/106186004x12632
8. Molnar V. Experimental Investigation of Tribology-Related Topography Parameters of Hard-Turned and Ground 16MnCr5 Surfaces. Lubricants 2023;11(6), doi:10.3390/lubricants11060263
9. Yang Y, Knust S, Schwiderek S, et al. Protein Adsorption at Nanorough Titanium Oxide Surfaces: The Importance of Surface Statistical Parameters beyond Surface Roughness. Nanomaterials 2021;11(2), doi:10.3390/nano11020357
